# Engineered biochemical cues of regenerative biomaterials to enhance endogenous stem/progenitor cells (ESPCs)-mediated articular cartilage repair

**DOI:** 10.1016/j.bioactmat.2023.03.008

**Published:** 2023-05-02

**Authors:** Liangbin Zhou, Jietao Xu, Andrea Schwab, Wenxue Tong, Jiankun Xu, Lizhen Zheng, Ye Li, Zhuo Li, Shunxiang Xu, Ziyi Chen, Li Zou, Xin Zhao, Gerjo J.V.M. van Osch, Chunyi Wen, Ling Qin

**Affiliations:** a Musculoskeletal Research Laboratory of Department of Orthopaedics and Traumatology & Innovative Orthopaedic Biomaterials and Drug Translational Research Laboratory of Li Ka Shing Institute of Health Sciences, Faculty of Medicine, The Chinese University of Hong Kong, 999077, Hong Kong SAR, China; b Department of Biomedical Engineering, Faculty of Engineering, The Hong Kong Polytechnic University, 999077, Hong Kong SAR, China; c Department of Orthopaedics and Sports Medicine, Erasmus MC, University Medical Center Rotterdam, 3015 GD, Rotterdam, the Netherlands; d Department of Oral and Maxillofacial Surgery, Erasmus MC, University Medical Center Rotterdam, 3015 GD, Rotterdam, the Netherlands; e Centre for Regenerative Medicine and Health, Hong Kong Institute of Science & Innovation, Chinese Academy of Sciences - CRMH, 999077, Hong Kong SAR, China; f Department of Biomedical Engineering, Faculty of Engineering, The Chinese University of Hong Kong, 999077, Hong Kong SAR, China; g Department of Otorhinolaryngology, Erasmus MC, University Medical Center Rotterdam, 3015 GD, Rotterdam, the Netherlands; h Department of Biomechanical Engineering, Faculty of Mechanical, Maritime and Materials Engineering, Delft University of Technology (TU Delft), 2600 AA, Delft, the Netherlands; i Centre for Translational Medicine Research and Development, Shenzhen Institute of Advanced Technology, The Chinese Academy of Sciences, 518000, Shenzhen, China

**Keywords:** Regenerative biomaterials, Endogenous stem/progenitor cells (ESPCs), Articular cartilage (AC) repair, Biochemical cues

## Abstract

As a highly specialized shock-absorbing connective tissue, articular cartilage (AC) has very limited self-repair capacity after traumatic injuries, posing a heavy socioeconomic burden. Common clinical therapies for small- to medium-size focal AC defects are well-developed endogenous repair and cell-based strategies, including microfracture, mosaicplasty, autologous chondrocyte implantation (ACI), and matrix-induced ACI (MACI). However, these treatments frequently result in mechanically inferior fibrocartilage, low cost-effectiveness, donor site morbidity, and short-term durability. It prompts an urgent need for innovative approaches to pattern a pro-regenerative microenvironment and yield hyaline-like cartilage with similar biomechanical and biochemical properties as healthy native AC. Acellular regenerative biomaterials can create a favorable local environment for AC repair without causing relevant regulatory and scientific concerns from cell-based treatments. A deeper understanding of the mechanism of endogenous cartilage healing is furthering the (bio)design and application of these scaffolds. Currently, the utilization of regenerative biomaterials to magnify the repairing effect of joint-resident endogenous stem/progenitor cells (ESPCs) presents an evolving improvement for cartilage repair. This review starts by briefly summarizing the current understanding of endogenous AC repair and the vital roles of ESPCs and chemoattractants for cartilage regeneration. Then several intrinsic hurdles for regenerative biomaterials-based AC repair are discussed. The recent advances in novel (bio)design and application regarding regenerative biomaterials with favorable biochemical cues to provide an instructive extracellular microenvironment and to guide the ESPCs (e.g. adhesion, migration, proliferation, differentiation, matrix production, and remodeling) for cartilage repair are summarized. Finally, this review outlines the future directions of engineering the next-generation regenerative biomaterials toward ultimate clinical translation.

## Highlights

• Numerous joint-resident ESPCs and chemokines, cytokines, and growth factors (CCGs) play critical roles in cartilage regeneration.
• Acellular regenerative biomaterials-based ESPCs-mediated cartilage repair exhibits several superiorities over endogenous cartilage healing and *in vitro*-manipulated cell-based therapies.
• Favorable exogenous biochemical cues have been shown to guide and magnify the roles of ESPCs.
• To date, we cannot conclude which biochemical cue or combination has the most stimulative effect due to the lack of standardized comparison in the preclinical studies.
• Combining cell-free regenerative scaffolds with engineered biochemical cues still represents one of the most promising strategies for cartilage repair.

## Graphical abstract

**Figure:**
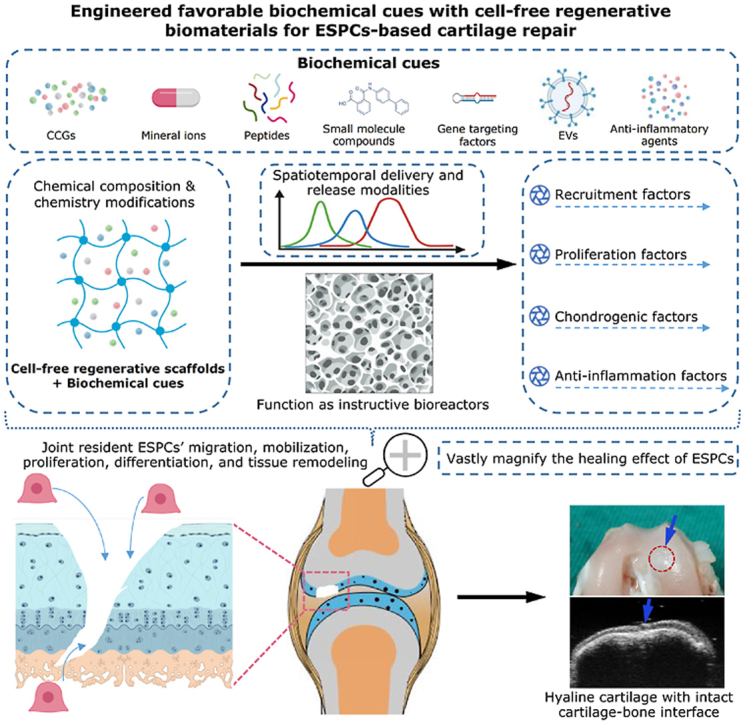

## Teaser

Statement of significance: Clinically, bone marrow stimulation techniques reinforce the healing effect of endogenous cartilage repair, which largely relies on activating the repairing effect of bone marrow-derived mesenchymal stem cells (BM-MSCs). Yet such techniques only offer insufficient and temporary improvement. Cytotherapy holds good promise for cartilage regeneration; however, troublesome details persist, such as donor-site morbidities, cell selection, viability, delivery, and phenotypic stability, apart from regulatory issues, timing of treatments, and prohibitive costs. Recently, a growing body of studies utilizing acellular regenerative implants have achieved desirable results, revealing biochemical cues’ multiplier effect on guiding and magnifying the roles of joint-resident ESPCs. Hereby, we summarize the latest scientific advances and cutting-edge technological innovations in the field of cartilage repair at the crossroad of endogenous cells and exogenous biochemical cues. We attempt to provide a new paradigm of cartilage repair with a focus on the enhanced healing effect of endogenous cells via exogenous biochemical cues toward the successful activation and maintenance of ESPCs-mediated cartilage repair. Our proposed strategy may represent one of the most promising options for cartilage regeneration.

## 1 Introduction

Articular cartilage (AC) is a smooth, avascular, and aneural connective tissue with unique composition and structure [1,2]. Its structure and function are mainly dependent on chondrocytes that control the turnover of extracellular matrix (ECM) and maintain homeostasis. It is located at the bone surface to provide a wear-resistant and load-bearing interface within synovial joints [2]. The poor intrinsic healing potential of AC usually leads to permanent functional impairment and osteoarthritis (OA) in the absence of adequate treatment [1,2]. There will be a growing number of young patients suffering from cartilage injuries caused by trauma in the coming decades. Nonsurgical treatments such as intra-articular hyaluronic acid (HA) injections and oral nonsteroidal anti-inflammatory drugs mainly focus on reducing clinical symptoms and preventing the progression of AC damage [3]. To regenerate neocartilage tissues in the lesion site, surgical interventions, such as microfracture, mosaicplasty, ACI, and MACI are proposed and extensively applied [4]. Through drilling small holes in the bone to a depth of around 2–4 mm at the injury site, arthroscopic microfracture is used, in part, to access the endogenous multipotent mesenchymal stem cells from the underlying bony region and promote their migration, proliferation, and chondrogenic differentiation; while ACI and MACI implant cultured chondrocytes-formed microtissues into the defect area under a natural or synthetic membrane via surgical procedures [4]. The above-mentioned surgical treatments (i.e. endogenous cartilage repair and cell-based therapies) have achieved varying degrees of success. On the other side, these approaches face several drawbacks, such as limited chondrocytes or cartilage sources, incapability to repair large-size AC defects, and the reconstructed tissue consisting of mechanically inferior fibrocartilage and integrates with surrounding cartilage incompletely, leading to poor resistance to shear forces and deterioration in a longer follow-up [5].

**Fig. 1:**
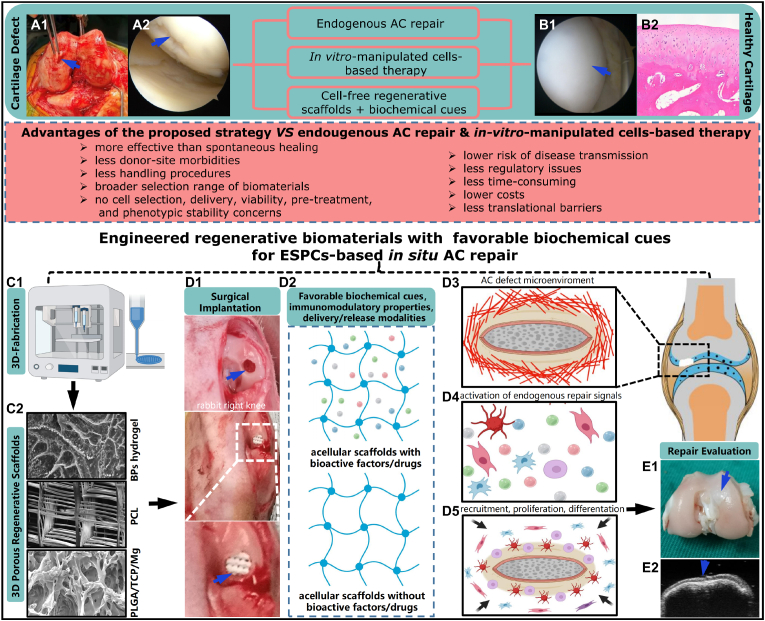
**Schematic diagram of the ESPCs-mediated cartilage repair strategies through 3D macro/micro-porous acellular engineered regenerative biomaterials.** (A1) Clinical photograph of an AC defect of the distal femoral condyle from the right knee of a 21-year-old male patient. (A2) Knee arthroscopic imaging of an advanced stage of AC defect of a 65-year-old female patient. (B1) An arthroscopy shows the smooth surface of healthy hyaline cartilage. (B2) The hematoxylin and eosin (HE) staining image indicates the unique hierarchical structure of the osteochondral unit including the upper AC. (C1) The fabrication of regenerative biomaterials by novel 3D-(bio)printing technologies. (C2) The scanning electron microscope (SEM) images demonstrate the porous architecture and desirable connectivity of the regenerative scaffolds (e.g. Bisphosphonates (BPs)-based hydrogel, PCL, and PLGA/TCP/Mg scaffolds). (D1) The surgical implantation of engineered regenerative biomaterials into the osteochondral lesion site (3 mm × 3 mm) in a rabbit model. (D2) The implanted acellular regenerative scaffolds (loaded with or without biomolecules, i.e., chemoattractants) which possess favorable biochemical cues, immunomodulation properties, and drug delivery/release profiles represent promising options for AC repair. (D3) Schematic illustration of the microenvironment around the cartilage defect. (D4) The activation of endogenous repairing signals. (D5) Possible illustration of the improved recruitment of numerous joint-resident ESPCs toward the lesion site by engineered regenerative scaffolds and enhanced proliferation and chondrogenesis of ESPCs, matrix production, and remodeling. (E1) One example of the engineered regenerative scaffolds for ESPCs-based AC repair: 12 weeks post-implantation into rabbits, the 3D-printed magnesium (Mg)-based acellular composite scaffold treatment improves to form smooth-surfaced cartilage, which has a similar hyaline-like appearance compared with adjacent AC tissue. (E2) The high-frequency ultrasound image shows the newly regenerated cartilage layer and cartilage-bone interface in the previous defect location. (A1, A2, and B1 images courtesy of Dr. Kevin Ki-Wai Ho and Dr. Yang Liu; C1, and D2-D5 were created by BioRender; Others are from the ongoing research project in our lab).

In recent decades, numerous studies have shown native ESPCs are involved in the complicated endogenous cartilage repair process, which is mainly dependent on the infiltration of these surrounding ESPCs into the cartilage lesions and subsequent cell behavior [6]. Without any exogenous interventions (e.g. allogeneic or xenogeneic cells transplantation, scaffolds implantation, and bioactive factors presentation and delivery), despite our body can rely on the inherent mechanism to recruit a few ESPCs, the capability of endogenous regeneration and repair is usually insufficient and incomplete, particularly in the longer term. For example, the clinical results of debridement and microfracture are inconsistent. The repaired tissue is predominantly fibrocartilage, which cannot be comparable to hyaline cartilage in terms of durability [7]. During neocartilage formation, aberrant collagen expression can be observed as a consequence of two different pathways, leading to the emergence of fibrocartilage (collagen I/II) or hypertrophic cartilage (collagen X). To regenerate hyaline cartilage (collagen II) both *in vivo* or/and *in vitro*, we should consider the strategies to provide low oxygen tension and suitable differentiation cocktails to induce chondrogenesis with less or no expression of collagen II and X [8]. In fact, the increased concentrations of chemokines, growth factors (GFs), and cytokines in tissues after AC injury is limited and last for a short period. Only a low number of ESPCs are recruited and able to function properly [9]. Meanwhile, with an in-depth understanding of the mechanism behind endogenous cartilage repair, various innovative cell-free regenerative biomaterial strategies have emerged as promising solutions for ESPCs-mediated cartilage regeneration [10,11] (Fig. 1). Acellular regenerative biomaterials-based ESPCs-mediated AC repair might be superior to exogenous cell-based therapeutic approaches in terms of handling procedures, accessibility of cell sources, donor-site morbidities, risk of disease transmission, costs, some regulatory issues, and translational barriers [12] (Fig. 1). In the scenario of ESPCs-mediated AC repair, regenerative biomaterials are defined as the scaffolds used to coax the body into recreating a pro-regenerative environment, influencing the immune system, and restoring the structure and function of damaged cartilage [[13], [14], [15]]. Despite more mechanistic studies being required, they are already poised to gain an immediate patient impact, representing an alternative paradigm for AC regeneration. Meta-analysis of *in vivo* animal studies indicated that implanting acellular regenerative biomaterials substantially enhanced AC repair by 15.6% compared with non-treated blank controls, i.e. endogenous cartilage repair [16]. Biologics supplementation could considerably improve AC regeneration by 7.6% in contrast to control scaffolds [16]. These results suggested cell-free engineered regenerative biomaterials with favorable biochemical cues could enhance ESPCs-mediated AC repair. Regenerative biomaterials usually act as instructive scaffolds to provide structural support for cell infiltration, matrix deposition, and tissue remodeling and regeneration (Fig. 1). Encouragingly, in March 2022 the FDA approved Agili-C™, a cell-free, off-the-shelf implant for repairing cartilage and osteochondral defects (OCD) [17], providing us with more confidence in our proposed strategy. From the scope of sources, three main types of regenerative scaffolds that are typically used for AC restoration, including natural biomaterials (e.g. cellulose, alginate, chitosan, gelatin, collagen, fibrin, chondroitin sulfate (CS), agarose, and HA), synthetic biomaterials (e.g. polyethylene glycol (PEG), polyvinyl alcohol (PVA), polycaprolactone (PCL), poly (lactic-co-glycolic acid) (PLGA), poly (propylene fumarates) (PPF), poly (NiPAAm), and polyurethane (PU)) and composite constructs [10]. Through different engineering methodologies of (bio)design and (bio)fabrication, these three-dimensional (3D) porous regenerative scaffolds can be functionalized with some tailored favorable biochemical cues, tunable chondro-immunomodulation, and various spatiotemporal delivery/release modalities [[18], [19], [20], [21], [22], [23], [24], [25], [26]] (Fig. 1). The injectable or implantable regenerative biomaterials (by themselves or combined with biomolecules) can kick-start and vastly magnify the body's intrinsic cartilage healing potential [9,27,28]. These biomaterials can bring a pro-regenerative microenvironment and take advantage of this friendly microenvironment as a natural bioreactor. Within this bioreactor, multiple stimuli derived from the regenerative scaffolds are capable of activating and recruiting a large population of join-resident ESPCs toward the lesion site, guiding their migration, mobilization, proliferation, and chondrogenesis to generate natural hyaline-like AC eventually [9,27,28] (Fig. 1). With huge translational potential, this strategy has attracted widespread attention and might represent one of the most promising therapies for chondral defects [29].

Unfortunately, most current proposed strategies for AC defects merely emphasize the regulation of a single healing period (i.e. cellular colonization), overlooking the integrity and continuity of distinct stages, which cannot provide an optimal solution for ESPCs-mediated AC repair. In this review, we emphasize all repair procedures rather than merely ESPCs migration. The ability to precisely control the regenerative scaffold-based *in vivo* microenvironment is still nascent. However, we feel that given the rapid progress in understanding the mechanism of endogenous AC healing and regenerative scaffolds, it is now the right time to discuss these issues and opportunities. This review mainly focuses on the engineered regenerative biomaterials-based approaches for guiding ESPCs for AC repair. In the first part, the potential mechanism of endogenous cartilage repair and the significance of ESPCs, chemokines, cytokines, and GFs (CCGs) will be discussed and summarized. Followed by a number of currently existing crucial challenges, various recent multidisciplinary achievements and advances in ESPCs-mediated strategies by manipulating the various amenable biochemical cues (e.g. chemical composition, biochemical modification, chemokines, cytokines and GFs, mineral ions, functional peptides, small molecule compounds, gene targeting factors, extracellular vesicles (EVs), immunomodulatory agents, and delivery/release profiles) of engineered regenerative biomaterials will be highlighted and discussed. The last part comprises conclusions and perspectives, accompanied by several critical open questions that still need to be addressed.

## 2 The vital roles of ESPCs, chemokines, cytokines, and growth factors (CCGs) for ESPCs-mediated AC repair

### 2.1 Endogenous cartilage healing and its possible mechanism

Intrinsic tissue regeneration capabilities are distinct among different species. Comparing with non-mammalian vertebrates, mammals and humankind possess limited inherent tissue self-healing capability due to genetic, developmental, immunologic, and tissue complexity differences [27,30]. For instance, the axolotl salamander (*Ambystoma mexicanum*) can heal large chondral defects and regenerate normal hyaline AC and joint structure even if limb amputation, whereas our human beings cannot [31]. Particularly noteworthy is that the endogenous cartilage repair potential decreases with aging, phylogeny, and ontogeny due to ESPCs exhaustion [32,33]. It implies that young and juvenile patients hold greater potential for endogenous cartilage healing than the elderly [34]. Unlike exogenous regenerative approaches, endogenous cartilage regeneration does not depend on exogenous cells, scaffolds, and biomolecules and only depends on the innate self-healing potential [35].

**Fig. 2:**
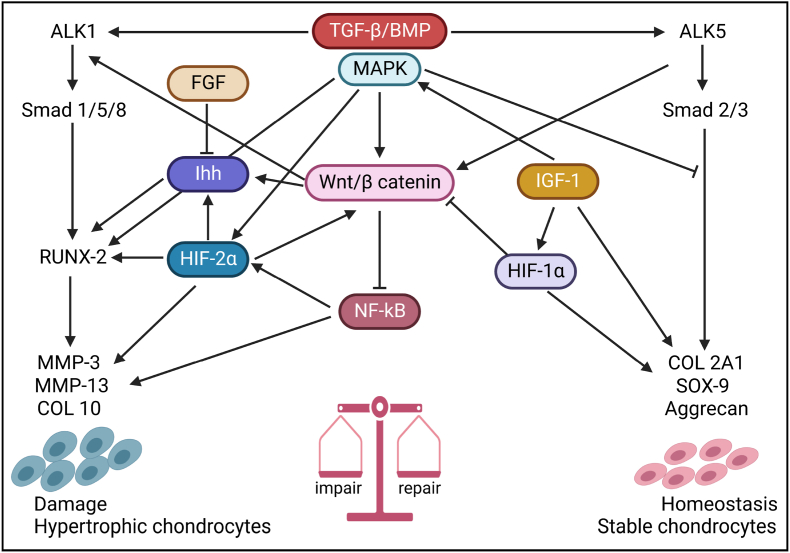
**The schematic illustration of the signaling crosstalk of cartilage tissue homeostasis and repair.** These signaling mainly comprise mitogen-activated protein kinase (MAPK), transforming growth factor-β (TGF-β), hypoxia-induced factors (HIF), bone morphogenetic proteins (BMPs), nuclear factor kappa B (NF-κB), Wnt/β-catenin, and indian hedgehog (Ihh) pathways, which control the balance driving for and catabolic and anabolic activities in AC (Adapted and reproduced from Mariani et al. [41], Copyright 2014, MDPI).

The typical repair process of AC defects is extremely complicated. It consists of a sequence of dynamic biological responses following a similar pattern, including hemostasis, inﬂammation, and remodeling stages (ESPCs recruitment from surrounding niches, proliferation, chondrogenesis, matrix deposition, and maturation) [36]. Under ideal conditions, these stages function coordinately with each other to assure the best repairing outcome. The presence of specific cells (e.g. immune cells, stem cells, and chondrocytes etc.) and vascular supply are the two prominent essential elements. After hemostasis, immune cells (e.g. neutrophils, macrophages, etc.) are recruited and activated by cytokines and chemoattractants secreted by the platelets [36,37]. Then immune cells can secrete some anti-inflammation factors and chondrogenic cytokines. This can further suppress inflammation and give rise to cellular exudation into the damaged area for fibrous network formation, which is invaded by ESPCs and chondrocytes during the remodeling phase, aiming to restore the original structure and function [37]. Therefore, some immune cells (i.e. macrophages) can act as a potential targets for AC repair [38]. The inflammation and remodeling phases rely on the vascular supply. Thus, compared to partial-thickness AC defects, the endogenous repair of full-thickness AC defects and OCD follow a different approach because of the participation of the vascular system from the lower subchondral bone [10,39]. The articular surface of full-thickness AC defects and OCD can self-repair without cell transplantation probably by recruiting endogenous cells from adjacent tissues and activating the autotherapy process [40]. This process is accompanied by inflammation and remodeling phases. However, the endogenous repair of partial-thickness AC defects is different due to the absence of a vascular system, limited inflammation, and insufficient chemokines and GFs. Moreover, the chondrocytes are imprisoned in glycosaminoglycans (GAGs) and collagens and are limited to migrate to the injured area from the surrounding cartilage. Thus, endogenous intra- and peri-articular ESPCs are even more vital in this context [40]. Therefore, joint-resident ESPCs from local or adjacent cell niches post-traumatically play a central role in endogenous cartilage healing. Maintaining homeostasis is finely tuned by a complicated network of signaling molecules and pathways (e.g. TGF-β, BMP, MAPK, Wnt/β-catenin, NF-κB, Ihh, HIF-1α, HIF-2α, IGF-1, and FGF) (Fig. 2). Studying endogenous cartilage repair and its underlying mechanism will help us understand how the human AC heals and repairs itself spontaneously. Additionally, it could assist researchers in developing innovative regenerative biomaterials as instructive bioreactors for guiding ESPCs to heal the AC more.

### 2.2 Joint-resident ESPCs and ESPCs-mediated AC repair

Cells are the building blocks for AC tissue engineering [10]. Many studies utilized *in vitro* manipulated cells and injected or implanted them into cartilage lesions, providing exogenous cell sources for neocartilage formation [42]. Compared with joint-resident ESPCs-mediated AC repair, these approaches result in challenges rooted in acquiring suitable high-quality, preferably sufficient autologous cells and rebuilding essential *in vitro* microenvironmental signaling that regulate *in vivo* tissue development and morphogenesis [10,43,44]. Besides, when using allogeneic or xenogeneic cells, the patients may need long-term immunosuppression therapies, probably impairing the treatment benefits. Moreover, these approaches usually ignore the donor's disease state and other features (e.g. age, ongoing chronic inflammation, and overall health conditions), perhaps influencing the tissue integration as well as the long-term survival of injected cells and engineered AC constructs [45].

**Fig. 3:**
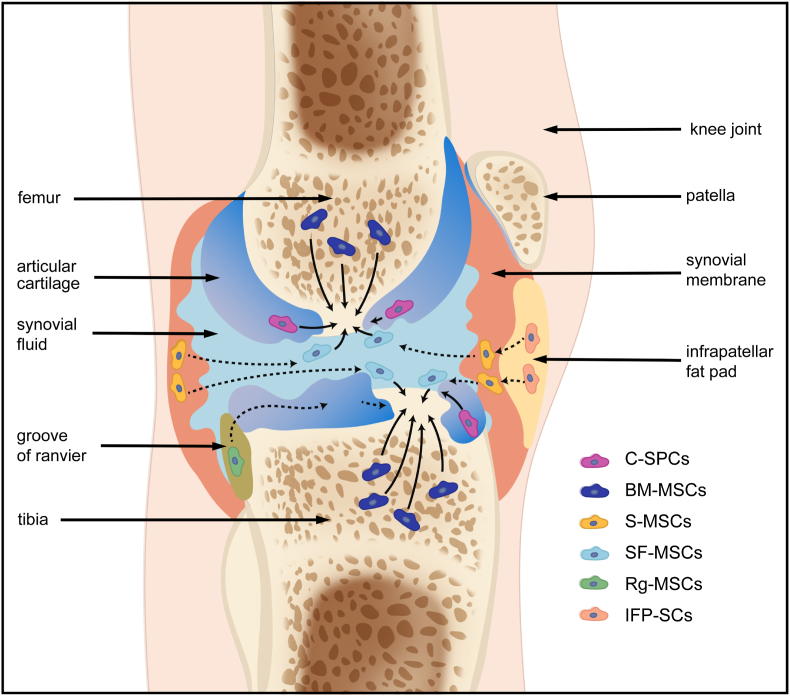
**The possible migration routes (PMRs) of native joint-resident ESPCs for AC repair.** Within the knee joint, there exist several different cell populations of ESPCs, including cartilage-derived C–SPCs, bone marrow-derived BM-MSCs, synovium tissue-derived S-MSCs, synovial fluid-derived SF-MSCs, ranvier groove-derived Rg-MSCs, infrapatellar fat pad- derived IFP-SCs, and so on. To date, there are huge knowledge gaps in the specific roles of different ESPCs during cartilage healing and the underlying mechanisms.

Here, we suppose that cell sources for AC repair should be poised for a paradigm shift from exogenous cells or *in vitro* manipulated autologous cells to joint-resident ESPCs thanks to the emergence of advanced technologies of shifting the injured microenvironment into a pro-regenerative environment with reduced inflammation and activation of endogenous repairing signals to some degree. The ‘endogeny’ portion highlights the induction of optimal endogenous AC healing by ESPCs; whereas reaching this goal needs exogenous intervention more or less, for example, implanting acellular engineered regenerative scaffolds can ameliorate the diseased microenvironment suffered chronic inflammation, low abundance of ESPCs, and dysregulated tissue turnover, into a pro-regenerative scenario [9,27,28]. ESPCs are tissue-specific adult stem/progenitor cells with self-renewal and differential abilities for maintaining AC homeostasis and repairing injured AC [9,27,28]. In recent decades, ESPCs have been identified and explored as eligible cell sources for *in vivo* AC regeneration [9,28]. Residing in specific niches of knee joints, ESPCs' activation relies on biophysical and biochemical cues within the niches. These niches are from AC and intra- or peri-articular tissues, such as bone marrow, synovial fluid, synovium, ranvier groove, fat pad, cartilage, subchondral bone, periosteum, and meniscus [28] (Fig. 3). Niches can provide ESPCs with instructive microenvironments that regenerative biomaterials can re-establish. Typically, a cell niche comprises ECM, cells, and soluble factors. ECM usually functions as a physical scaffold for signaling molecules and cells and is a major regulator and determinant of stem cell fate [46]. Within the ECM, various secreted proteins interact with resident cells dynamically. Distinct cell receptors (e.g. cadherins and integrins) can mediate cell-ECM interactions. Receptors are crucial adhesion molecules for ESPCs' migration, localization, survival, and differentiation.

Different subpopulations of ESPCs possess varied surface markers and chondrogenic differentiation abilities (as indicated in Table 1). For example, synovium-derived MSCs (S-MSCs) have been reported to possess the optimal chondrogenic capacity *in vitro* with a lower potential for hypertrophy among the mesenchymal tissue-derived cells [47,48]. Many studies have provided evidence of the recruitment and migration of ESPCs for cartilage repair *in vivo* [[49], [50], [51]]. Ma et al. (bio)fabricated the macro-porous SA/HA_exo_-PLGA_KGN_ hydrogel scaffolds which exhibited desirable results of regulating inﬂammation homeostasis and recruiting endogenous bone marrow mesenchymal stem cells (BM-MSCs) for AC repair in rats via the sequentially deliver of LPS/BG-exo and Kartogenin (KGN) [52]. Huang et al. injected the BM-MSCs affinity peptide sequence PFSSTKT (PFS)-modified chondrocyte ECM particles combined with methacrylated gelatin (GelMA) hydrogel into a rabbit cartilage defect model [53]. The results showed the GelMA/ECM-PFS functional scaffolds promoted the recruitment of ESPCs from the defect site two weeks post-operation and generated hyaline cartilage *in vivo*, whereas the control treatment mostly led to fibrocartilage formation. The possible migration routes (PMRs) of joint-resident ESPCs for AC repair are graphically displayed in Fig. 3. There might be different ESPCs involved in the repairing process depending on the category of chondral damages [28]. For adults, BM-MSCs can make direct contributions to regenerating full-thickness AC defects. Yet it remains unclear how BM-MSCs migrate to the superficial area. Additionally, experimental evidence has confirmed the direct migration of SF-MSCs, S-MSCs, and C–SPCs to superficial chondral defects. IFP-SCs may function after being recruited toward the adjacent synovial fluid and synovial lining. The PMR of Rg-MSCs along the perichondrium has also been explored in rabbit knee joints.

**Table 1:** Subpopulations and characteristics of native joint-resident ESPCs.

Cell Types	Location	Specific positive surface markers	Chondrogenic potential and effects on AC repair	Year of first reported
BM-MSCs	Perivascular niches in bone marrow	CD29*^^^, CD44*^&^, CD73*^&^, CD90*^&^, CD105*^&^, CD147*^^^, CD166*^^^, CD271*^&^	Multilineage potential includes chondrogenesis [54]; however, they hold a high tendency to cause hypertrophic chondrocytes and bone formation [55]. CD271+ CD56^+^ BM-MSCs (localized in the bone-lining regions) have a better chondrogenic capacity compared to CD271+ CD56^−^ BM-MSCs (found in the perivascular regions) [56].	1969
S-MSCs	Synovium of joint	CD10*^&^, CD13*^&^, CD14*^&^, CD34*^&^, CD44*^&^, CD45*^&^, CD49a*^&^, CD62e*^&^, CD73*^&^, HLA-DR*^&^, CD90*^&^, CD105*^^^, CD147*^^^, CD166*^^^	Reported as the best chondrogenesis potential among mesenchymal tissue-derived cells [47]. Limited potential for hypertrophy compared to BM-MSCs, IFP-SCs, and SM-MSCs [48]. CD73^+^CD90^−^ S-MSCs have a better chondrogenic capacity compared to CD73^+^CD90^+^ S-MSCs [57].	2001
SF-MSCs	Synovial fluid of joint	CD40^#&^, CD44*^^^, CD44*^&^, CD55*^&^, CD73*^&^, CD90*^&^, CD105*^&^, CD140*^&^, CD147*^^^, CD273*^&^	High capacity to differentiate into chondrocytes, and a lower capacity for adipogenic, osteogenic, and neurogenic differentiation [58].	2004
Rg-MSCs	Perichondrial groove of ranvier	Stro-1*^&^, BMPr1a*^&^, Patched*^&^, Notch1*^&^, integrin β1*^&^, N-cadherin*^&^, EGFL7*^&^	They can maintain their progenitor properties and localization and migrate to the AC surface [59].	1977
IFP-SCs	Intra-articular fat pad	CD9*^^^, CD10*^^^, CD13*^^^, CD29*^^^, CD44*^^^, CD49*^^^, CD59*^^^, CD90*^^,^ CD105*^^^, CD104*^^^, CD105*^^^, CD147*^^^, CD166*^^^	They can maintain their chondrogenic potential for a longer period [60]. A better chondrogenic potency compared with BM-MSCs.	1996
C–SPCs	Mainly in the superficial zone of AC	CD29*^^^, CD44*^^^, CD54*^^^, CD73^#^^, CD90^#^^, CD105*^&^, CD166*^&^, Stro-1*^^^, Notch-1^#^^	Superficial cells of the nascent joint are self-renewing chondrocyte progenitors and undergo both symmetric and asymmetric cell division [61]; Stronger chondrogenic differentiation capacity than the IFP-SCs and chondrocytes [62]; Cells migrate during the development and remodeling of AC [63].	2001
CS-PCs	Subchondralcancellous bone	CD44*^^^, CD73*^^^, CD90*^^^, CD105*^^^, CD166*^^^	They showed chondrogenic differentiation potential [64].	2008
M-SPCs	Meniscus red zone	CD29*^&^, CD44*^&^, CD73^#^^, CD90*^&^, Sca-1^#^^, CD105*^&^, CD166*^^^	Comparable chondrogenic potential to C–SPCs [65];	2009
SM-MSCs	Muscle	NGFR*^^^, CD44*^^^, CD49e*^^^, CD73*^^^, CD90*^^^, CD105*^^^, CD147*^^^, CD54*^^^, CD166*^^^	SM-MSCs harvested from traumatized muscle display a similar phenotype to BM-MSCs [66].	1961
P-MSCs	Periosteum	CD10*^^^, CD44*^^^, VEGFR-2*^^^, CD10*^^^, CD44*^^^, CD54*^^^, CD90*^^^, CD105*^^^, CD147*^^^, CD166*^^^	The similar multipotency to BM-MSCs [67]; Highest calcification potential compared to BM-MSCs, S-MSCs, IFP-MSCs, and SM-MSCs [68].	1990

S-MSCs: synovium-derived MSCs; SF-MSCs: synovial fluid-derived MSCs; Rg-MSCs: MSCs in the groove of Ranvier; IFP-SCs: Intra-articular fat pad-derived stem cells; C–SPCs: cartilage-derived stem/progenitor cells; CS-PCs: Cortico-spongious progenitor cells; M-SPCs: meniscus stem/progenitor cells; P-MSCs: periosteum-derived MSCs; SM-MSCs: skeletal muscle-derived MSCs; *: characterized on human-derived tissue/primary cells; #: characterized on animal-derived tissue/primary cells; &: characterized on tissue; ^: characterized on expanded cells *in vitro*.

### 2.3 CCGs for ESPCs-mediated AC repair

The migration of ESPCs is a prerequisite for endogenous AC repair [9]. Many CCGs are involved in the complicated process of recruiting ESPCs from their previous niches. GFs are polypeptide extracellular signaling molecules that play vital roles in regulating cell migration, proliferation, differentiation, and survival [9,69]. Numerous GFs function synergistically to regulate AC development and homeostasis. The expression of GFs by chondrocytes is increased after injury [70,71]. In recent years, several GFs, such as insulin-like growth factor (IGF), platelet-derived growth factor (PDGF), and TGF-β have been intensively explored for their physiological effects on chondral repair both *in vitro* and *in vivo* [[72], [73], [74]]. Chemokines are small proteins (8–10 kDa) expressed in tissues in response to injury or infection. On the basis of the number and spacing of cysteine residues, they can be categorized into four subfamilies: CC, CXC, XC, and CX3C [75]. ESPCs can be attracted by the activation of chemokines to migrate along the chemotactic gradients and are involved in various following repair stages [75]. ESPCs express various receptors for chemokines, such as CXC chemokine receptors 1 and 2 (CXCR1 and CXCR2), CC chemokine receptor 1 (CCR1) and CCR2, and receptors of GFs such as PDGF receptors a (PDGFR-a) and b (PDGFR-b). Besides, inflammatory cytokines are crucial for regulating the inflammation balance of the defect site. The detailed information of CCGs regarding the members and the potential regulatory effects for ESPCs-mediated cartilage repair are listed in Table 2.

**Table 2:** The effects of endogenous CCGs on guiding ESPCs for AC regeneration.

Guiding factors	CCGs	Family members	Regulatory effects	Reference
Recruitment factors	chemokines	CCL2 (MCP-1), CCL5 (RANTES), CCL17 (TARC), CCL19 (MIP-3β), CCL20 (MIP-3α), CCL21 (SLC), CCL22 (MDC), CCL25 (TECK), CCL28 (MEC), CXCL7 (NAP-2), CXCL8 (IL-8), CXCL10 (IP-10), CXCL11 (I-TAC), CXCL12 (SDF-1), CXCL13 (BLC), CXCL16 (SR-PSOX), XCL1 (Lptn)	To stimulate the chemotaxis of ESPCs	[9,28,29]
	GFs	PDGF-AA, PDGF-AB, PDGF-BB, IGF-1, IGF-2, IGFBP-5, TGF-β1, TGF-β3, BMP-2, BMP-4, BMP-7, VEGF-A, FGF-2, HGF, EGF, HB-EGF		
Proliferation factors	GFs	IGF-1, TGF-β1, TGF-β3, BMP-2, BMP-4, BMP-7, TGF-β, FGF-2, FGF-9, FGF-18	To stimulate the cell proliferation of ESPCs	[9,76]
Differentiation factors	GFs	TGF-β1, TGF-β3, BMP-2, BMP-6, BMP-7, FGF-9, FGF-18, Ihh, PTHrP, Wnt-4, Wnt-8, VEGF	To stimulate ESPCs' chondrogenesis	[9,76]
Inflammatory factors	cytokines	TGF-β, IL-10, IL-4 (anti-inflammation)	To modulate the inflammatory balance	[77]
		IL-1β, IL-6, TNF-α, IL-8,IL-17, IL-18, IFN-γ (pro-inflammation)		

MCP-1: monocyte chemoattractant protein-1; RANTES: regulated on activation, normal T cell expressed and secreted; TARC: thymus- and activation-regulated chemokine; MIP: macrophage inflammatory protein; SLC: secondary lymphoid-tissue chemokine; MDC: macrophage-derived chemokine; TECK: thymus-expressed chemokine; MEC: mucosae-associated epithelial chemokine; LEC: liver-expressed chemokine; CTACK: cutaneous T-cell attracting chemokine; PARC: pulmonary and activation-regulated chemokine; NAP-2: neutrophil-activating peptide; IL-8: interleukin-8; IP-10: interferon-inducible protein-10; I-TAC: interferon-inducible T cell alpha chemoattractant; SDF-1: the stromal cell-derived factor-1; BLC: B lymphocyte chemoattractant; SR-PSOX: scavenger receptor for phosphatidylserine and oxidized lipoprotein; ENA-78: epithelial-derived neutrophil-activating peptide; GRO-α: growth-regulated oncogene-alpha; LPtn: lymphotactin; PDGF: platelet-derived growth factor; IGF: insulin-like growth factor; TGF: transforming growth factor; BMP: bone morphogenetic protein; VEGF: vascular endothelial growth factor; FGF: fibroblast growth factors; HGF: hepatocyte growth factor; EGF: epidermal growth factor; HB-EGF: Heparin-binding-epidermal growth factor.

## 3 Macro/micro-porous regenerative scaffolds function as instructive bioreactors for ESPCs and their current challenges for EPSC-based AC repair

More recently, macro/micro-porous regenerative biomaterials-based therapy has evolved as a potentially powerful paradigm in cartilage regenerative medicine [78,79]. Typically, these cell-free scaffolds, serving as instructive bioreactors of ESPCs, can promote ESPCs-mediated AC repair on their own or in combination with biologics. With optimized biochemical and biophysical cues, they can be fabricated into varied shapes, sizes, and formulations [15]. These cues play fundamental roles in providing a pro-regenerative microenvironment, open porous structures allowing for coaxing the directional cell homing and infiltration of ESPCs, and supporting cell adhesion, proliferation, and chondrogenesis [15]. For example, the study from Levinson et al. demonstrated that adhesive HA–transglutaminase (HA-TG) hydrogel with chondrogenic properties in a collagen scaffold could allow for ESPCs invasion and promote ESPCs-mediated cartilage repair in an ovine model [80]. The ideal regenerative scaffolds should possess non-toxic, non-immunogenic, and satisfactory biocompatible and biodegradable properties [13,32]. They should be easily manufactured and ease in handling [15]. In the past decades, a plethora of regenerative scaffolds has been (bio)fabricated and assessed for AC repair in the form of bioglasses [81], sponges [82], hydrogels [11], electrospun fibers [83], micro/nanoparticles [49,84], etc. An overview of the pros and cons, as well as specific applications for cartilage repair of various regenerative biomaterials is summarized in another review from Duarte Campos et. al [14]. Naturally-derived biomaterials have been demonstrated several advantages compared to synthetic biomaterials: They hold better biocompatibility, biodegradability, and remodeling properties compared to synthetic biomaterials [14]. For example, animals or human-derived collagen and fibrin consist of cell adhesion ligands and can be vulnerably proteolytically cleaved and degraded, enabling cell infiltration and remodeling. These scaffolds interact with cells by specific surface ligands, contributing to ESPCs migration, proliferation, and matrix deposition [15]. Synthetic biodegradable polymers (e.g. PCL, PLA, PLGA, PLLA, PVA, and PEG) offer some advantages over natural materials, including high reproducibility, controlled degradation rate *in vivo*, easy manipulation into specific shapes, and high mechanical strength; nevertheless, such scaffolds lack the cell recognition signals [14,15]. Thus, synthetic scaffolds are often modified with proteins and peptides to support ESPCs infiltration. Containing two or more different constituent biomaterials or phases on a microscopic or macroscopic size scale, a composite biomaterials consisting of natural and synthetic materials can combine the advantages of synthetic polymeric materials with that of natural materials to achieve excellent mechanical properties, bio-functionality, and tunable degradability. Even though tremendous progress in AC repair has been achieved by synthetic and composite regenerative scaffolds, only a few of these scaffolds are now in clinical use or practice. The commercially available biomaterial products for AC repair are still primarily based on natural biomaterials such as collagen (MaioRegen Chondro^+^), HA (Chondrotissue® and Hyalofast®), and fibrin glue (Tisseel®) [10,85].

**Fig. 4:**
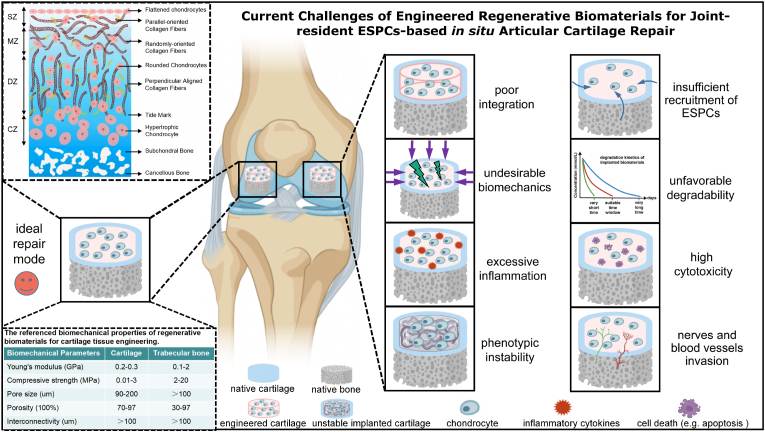
**Current challenges of engineered regenerative biomaterials-based guiding of ESPCs for cartilage repair.** Eight major challenges faced from the bench to beside include poor integration with adjacent cartilage, undesirable biomechanics for joint locomotion, excessive inflammatory environment, phenotypic instability over a longtime window, insufficient recruitment of ESPCs, unfavorable degradable characteristics, high cytotoxicity as well as nerves and blood vessels invasion. For the ideal repair mode, the implanted regenerative scaffolds should possess various favorable biochemical cues coupled with biophysical support to promote neocartilage formation whose both anatomical structure and biomechanical characteristics are comparable with surrounding healthy hyaline cartilage. (Partially created by BioRender. The diagram of AC stratified structure is reproduced from Zhou et al. [10], Copyright 2020, John Wiley and Sons).

From the scope of preclinical studies, challenges in AC repair often arise after the implantation of engineered regenerative biomaterials into defects. Poor integration with adjacent tissues, undesirable biomechanics for joint locomotion, excessive inflammatory environment, phenotypic instability in the longer run, insufficient recruitment of ESPCs, unfavorable degradable characteristics, high cytotoxicity as well as nerves and blood vessels invasion [2,10,86] impede the further translational potentials of these regenerative biomaterials (Fig. 4). To address the above limitations and challenges of current regenerative biomaterials, considerable efforts have been made to reinforce the integration with native cartilage or/and bone, achieve desirable biomechanics, improve anti-inflammation and immunity control, maintain cartilaginous phenotype stability, recruit and guide enough ESPCs, possess favorable degradability, increase biocompatible properties, and seek for anti-angiogenesis coupling with anti-neurogenesis strategies [9,11,28,87]. Some of them have achieved desirable preclinical results. However, regarding the ultimate clinical translation of the established optimal regenerative scaffolds-based ESPCs-mediated cartilage repair, it still has a long way to move forward.

## 4 Regenerative implants with favorable biochemical cues magnify the healing effect of ESPCs for AC repair

**Fig. 5:**
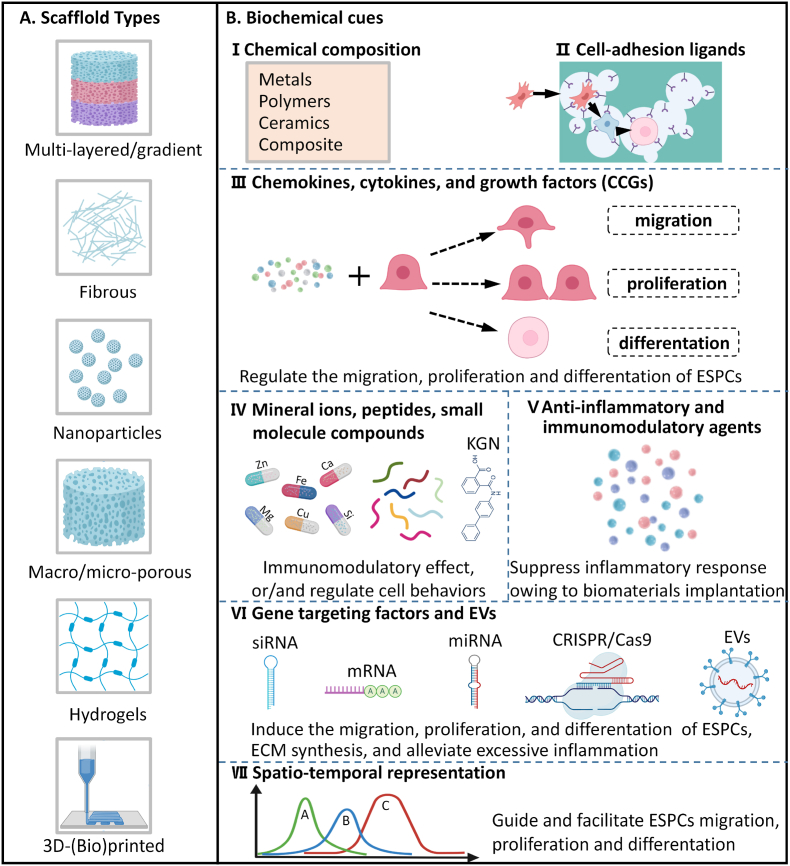
**Innovative (bio)design and (bio)fabrication of regenerative biomaterials with favorable biochemical cues to guide joint-resident ESPCs for AC repair.** (A) Several examples of regenerative scaffolds, including multi-layered/gradient scaffolds, fibrous scaffolds, nanoparticles, microporous scaffolds, hydrogels, and 3D-printed scaffolds, have been widely explored to harness the innate regenerative ability of cartilaginous tissues in preclinical studies. (B) Engineering regenerative scaffolds with appropriate biochemical cues through (Ⅰ) chemical composition and (Ⅱ) surface/interface chemistry modification of biomaterials to produce cell-adhesion ligands. These biochemical cues mainly include (Ⅲ) CCGs, (Ⅳ) peptides, mineral ions, and small-molecule compounds, (Ⅴ) anti-inflammatory and immunomodulatory agents, (Ⅵ) gene targeting factors and EVs (mRNA, messenger RNA; miRNA, microRNA; siRNA, small interfering RNA; and extracellular vesicles, EVs). The combination of regenerative scaffolds and engineered biochemical cues are usually presented as (Ⅶ) spatiotemporal delivery/release modalities. (Created by BioRender).

Due to their intrinsic characteristics, traditional biomaterials have shown limited capabilities in promoting cell recruitment, proliferation, and differentiation. Moreover, traditional biomaterial treatment might bring inadequate cartilaginous matrix deposition and maturation, a lack of natural anisotropic structures, and excessive inflammation [11,14]. However, advanced regenerative scaffolds with optimized biophysical and biochemical properties can overcome the above-mentioned challenges to some degree. It has been shown that biophysical and biochemical cues function synergistically to facilitate AC regeneration [88]. In this review, we only focus on tunable biochemical messages. Across the intracellular and extracellular environment, the gradient presence of biochemical cues is able to respond to multiple cell functional requests [9,88]. Many exogenous biochemical cues can be incorporated into biomaterials to regulate ESPCs' physiological activities, i.e. enhancing cell migration. Therefore, we think that an exquisite (bio)design and (bio)fabrication of regenerative scaffolds with appropriate biochemical cues holds the potential to guide ESPCs-mediated cartilage repair. A variety of multi-layered/gradient, fibrous, nanoparticle, macro/micro-porous, and hydrogel scaffolds have been constructed through many emerging cut-edging technologies and concepts including 3D-(bio)printing [49,52,[89], [90], [91]] (Fig. 5A). Their beneficial biochemical signals are usually rooted in chemical composition (Fig. 5B (Ⅰ)), (surface/interface) biochemical modification (Fig. 5B (Ⅱ)), CCGs (Fig. 5B (Ⅲ)), mineral ions, peptides, small molecule compounds (Fig. 5B (Ⅳ)), gene-targeting factors and EVs (Fig. 5B (Ⅴ)), anti-inflammatory and immunomodulatory agents (Fig. 5B (Ⅵ)), and spatiotemporal scaffold-based drug delivery systems (SDDS) (Fig. 5B (Ⅶ)). Some specific examples are listed below in Table 3. Novel regenerative scaffolds should ideally possess one or more features beyond conventional biomaterials, offering a pro-regenerative microenvironment for ESPCs’ homing and chondrogenesis as well as matrix production and maturation, and responding to dynamic changes in the environment throughout the neocartilage formation.

**Table 3:** Examples of engineered regenerative biomaterials with various favorable biochemical cues to guide ESPCs for AC repair.

Engineered biochemical cues	Specific examples	Other biologics & biomaterials	*In vitro/vivo*	Influences on SPCs or/and potential applications for ESPCs-mediated AC repair	Reference
Chemical composition	Composition ratios of Gel/HA hybrid hydrogels	N/A	*in vitro*	Different chemical composition ratios of Gel/HA hybrid hydrogels affected cell adhesion and chondrogenesis. The Gel/HA composite hydrogel (30%/70%) seemed the most promising matrix for chondrogenesis with balanced cell proliferation and adhesion.	[18]
	Li incorporation	Li_2_Ca_4_Si_4_O_13_ bioceramic	*in vitro*	A lithium-containing biomaterial promoted chondrogenesis of iPSCs with reduced hypertrophy.	[92]
	Composition ratios of PEG:CS:MMP-pep	N/A	*in vitro*	Unique biomaterial compositions (PEG:CS:MMP-pep) directed BM-MSCs into specific chondrocyte phenotypes correlating with distinct layers of AC.	[93]
(Surface/interface) chemical modification	Hydrophilic coating	PLGA scaffold	*in vitro*	The hydrophilic surface of biomaterials had beneficial effects on chondrocyte activity and matrix synthesis.	[94]
	Gelatin, collagen, chitosan coating	PLLA membrane	*in vitro*	PLLA membrane surfaces modified with natural macromolecule layers could enhance chondrocyte attachment, proliferation rate, and cell activity.	[95]
	HA modification	PLGA scaffold	*in vitro*	HA-modified PLGA scaffolds and HA-coated wells could improve the chondrogenesis of human ADSCs.	[96]
	HA modification	PGA scaffold	*in vitro*	HA coating of PGA scaffolds could significantly improve biocompatibility and cartilage formation.	[97]
	Hydrophilic coating	PLLA scaffold	*in vitro*	Hydrophilic coating using two or more natural macromolecules (CS and Collagen) on scaffolds may synergistically enhance chondrogenesis.	[98]
	CS surface grafting	PLLA fiber	*in vivo*; rabbit model	An aligned PLLA fiber scaffold grafting with a biomimetic CS surface for accelerating cartilage repair	[19]
	NB coating	SF microparticle	*in vivo*; rabbit model	JS-Paint, mainly formed by NB-coated SF microparticles, showed excellent properties for improving cell adhesion, migration, and proliferation which were critical for AC regeneration.	[99]
Chemokines, Cytokines, and GFs (CCGs)	CXCL12	fibrin/HA hydrogel	*ex vivo*; bovine OC explant	CXCL12-loaded fibrin/HA hydrogels could promote the functional repair of full-thickness AC defects through the homing of endogenous chondrogenic progenitor cells.	[100]
	IL-8- and MIP-3α	PLA/β-TCP scaffold	*in vivo*; beagle model	IL-8 and MIP-3α markedly improved the chemotaxis of BM-MSCs *in vitro*. IL-8- and MIP-3α-containing biomaterials recruited ESPCs for knee AC regeneration.	[101]
	IL-4 and IL-13	Gelatin/genipin microspheres	*in vitro*	Exposure to the IL-13 and IL-4 loaded microspheres alleviated the inflammation of chondrocytes up to 80%. The microsphere format allowed for minimally invasive delivery of anti-inflammatory cytokines for AC repair.	[102]
	IL-4	GelMA/PCL-HA scaffold	*in vivo*; rabbit model	The upper layer with IL-4 reduced the adverse inflammation effects on chondrocytes. IL-4-containing bi-layer scaffolds could promote the repairing of both AC and subchondral bone.	[103]
	TGF-β3	PLCL scaffold	*in vivo*; nude mice model	TGF-β3 encapsulated PLCL scaffold could help to yield hyaline cartilage-specific lacunae structures and prevent hypertrophic chondrocyte formation.	[104]
	TGF-β1	HA/HAp/PEG-PCL scaffold	*in vivo*; rabbit model	TGF-β1 containing composite scaffolds could improve the healing of cartilage and subchondral bone through improved effects on ESPCs adhesion, proliferation, and differentiation.	[20]
	PDGF-BB and TGF-β3	HAMA/HepMA microgel	*in vivo*; rat model	Stem cell-recruiting injectable microgels encapsulated with PDGF-BB and TGF-β3 for repairing cartilage.	[73]
	PRPs	PLPMH scaffold	*in vivo*; rabbit model	PRP-loaded macro-porous hydrogel scaffolds recruited endogenous M2 macrophages in large numbers and long-time duration (42 days) to support a local anti-inflammatory microenvironment for AC repair.	[51]
Mineral ions	Mg^2+^	N/A	*in vitro/vivo;* rabbit model	Mg^2+^ enhanced the adherence and cartilage formation of S-MSCs through integrins; Mg^2+^ enhanced the chondrogenesis of MSCs by inhibiting activated macrophage-induced inflammation.	[87,105]
	Mg^2+^	Mg-Nd-Zn-Zr alloy@PDA	*in vitro*	The Mg-based scaffolds could recruit MSCs, enhance chondrogenesis, attenuate local inflammatory responses by improving M2 macrophage polarization and down-regulating NF-κB signaling.	[21]
	Sr^2+^, Cu^2+^, Mn^2+^, Zn^2+^, Si^4+^	N/A	*in vitro/vivo*	Strontium, copper, manganese, zinc, and silicon-based scaffolds could improve cartilage formation.	[81,[106], [107], [108], [109]]
Chondroinductive/chondroconductive peptides	CK2.1	β-GP/CS-HAp/CS	*in vivo*; rabbit model	CK2.1-coated β-glycerophosphate chitosan composite scaffolds could promote AC repair in rabbits through the recruitment and induced chondrogenesis of ESPCs.	[23]
	PFSSTKT	dECM/RAD peptide	*in vivo*; rabbit model	Increased recruitment of ESPCs and chondrogenic differentiation by a composite scaffold loaded with bone marrow homing peptides for repairing AC.	[110]
	GGGHAVDI	NC/PdBT/GHK	*in vivo*; rabbit model	Hydrogels containing a chondrogenic peptide sequence could obtain higher histological assessments of overall defect filling, GAGs, cell contents, and cartilage surface regularity.	[89]
	DHLSDNYTLDHDRAIH	N/A	*in vitro*	Link protein N-terminal peptide significantly enhanced migration and chondrogenesis of SPCs *in vitro*.	[111]
	Ec peptide	TGF-β1	*in vitro*	Ec could facilitate *in vitro* hMSC mobilization and chondrogenesis and enhance the role of TGF-β1.	[112]
Small molecule compounds	Dexamethasone	PLGA/agarose	*in vivo*; canine model	Sustained delivery of low-dose dexamethasone (up to 99 days) by a PLGA microsphere-embedded agarose implant to attenuate inflammation and improve pro-anabolic effect for AC repair.	[113]
	KGN	SDF-1/PLGA/HA	*in vivo*; rabbit model	A cell-free therapy for AC defects via the synergistic delivery of SDF-1 & KGN (more than two months) within HA injectable hydrogels.	[22]
	Icariin	N/A	*in vivo*; rabbit model	Icariin promoted proliferation and chondrogenic differentiation of BM-MSCs *in vitro* and rabbit knee AC repair via the BMP/Smad pathway.	[114]
Gene targeting factors and EVs	antimiR-221	fibrin/HA	*ex vivo*; bovine OC explant	Hydrogel loaded with antimiR-221/lipofectamine could drastically enhance AC regeneration through ESPCs.	[24]
	miR-29b-5p	(SKPPGTSS) SAP hydrogel	*in vivo*; mice model	Sustained hydrogel-based delivery of miR-29b-5p could promote the recruitment and subsequent chondrogenic differentiation of endogenous cells, which were crucial for successful AC repair and chondrocyte rejuvenation.	[115]
	hWJMSC-Exos	dECM scaffold	*in vivo*; rat/rabbit model	hWJMSC-Exos could improve cell proliferation, migration, and polarization *in vitro*. hWJMSC-Exos injection could inhibit inflammation within the joint cavity and improve AC repair.	[116]
	DNA aptamer	SF/HA-Tyr hydrogel	*in vivo*; rabbit model	Apt19S-functionalized bilayer scaffold could dramatically enhance BM-MSCs migration *in vitro* and support AC repair by recruiting ESPCs toward the defect sites of rabbits.	[117]
	rAAV vector	PEO–PPO–PEO hydrogel	*in vivo*; minipig model	The PEO-PPO-PEO poloxamers-based thermosensitive hydrogels allowed for a controlled *in situ* release of rAAVs to repair chondral defects effectively.	[118]
Anti-inflammatory & immunomodulatory agents	Celebrex	N/A	*in vivo*; rat model	Celecoxib acted as chondroprotective and anti-inflammatory effects on AC both *ex vivo* and *in vivo*.	[119]
	Squid collagen II	N/A	*in vivo*; rat model	Squid collagen II promoted cartilage repair via inhibiting apoptosis and hypertrophy of chondrocytes and immunomodulating activation of M2 macrophages.	[120]
	GM-HPCH + TGFβ1	N/A	*in vivo*; rat model	The GM-HPCH + TGFβ1 hydrogels effectively improved AC repair by immunoregulating macrophages, recruiting ESPCs, and facilitating chondrogenesis.	[25]
	PRP-GelMA	N/A	*in vivo*; rabbit model	20% of PRP-GelMA hydrogels improved the chemotaxis and chondrogenesis of ESPCs, immune regulation, and macrophage polarization shift from M1-to-M2, which were suitable for AC repair.	[91]

MMP-pep: matrix metalloproteinase-sensitive peptides; NB: *N*-(2-aminoethyl)-4-(4-(hydroxymethyl)-2-methoxy-5-nitrosophenoxy) butanamide; HAp: hydroxyapatite; KGN: kartogenin; HAMA/HepMA: methacrylated HA and heparin; PLPMH: platelet lysate-rich plasma macro-porous hydrogel; β-GP: β-glycerophosphate; OC: osteochondral; PRP: platelet-rich plasma; SAP: self-assembling peptide; silk fibroin: SF; NC/PdBT/GHK: N-cadherin/poly(glycolic acid)-di(but-2-yne-1,4-dithiol)/glycine-histidine-lysine; hWJMSC-Exos: human umbilical cord Wharton's jelly MSC-derived exosomes; rAAVs: recombinant adeno-associated virus; PEO-PPO-PEO: poly(ethylene oxide)–poly(propylene oxide)–poly(ethylene oxide); GM-HPCH: glycidyl methacrylate-modified hydroxypropyl chitin.

### 4.1 Chemical compositions and chemistry modifications

**Fig. 6:**
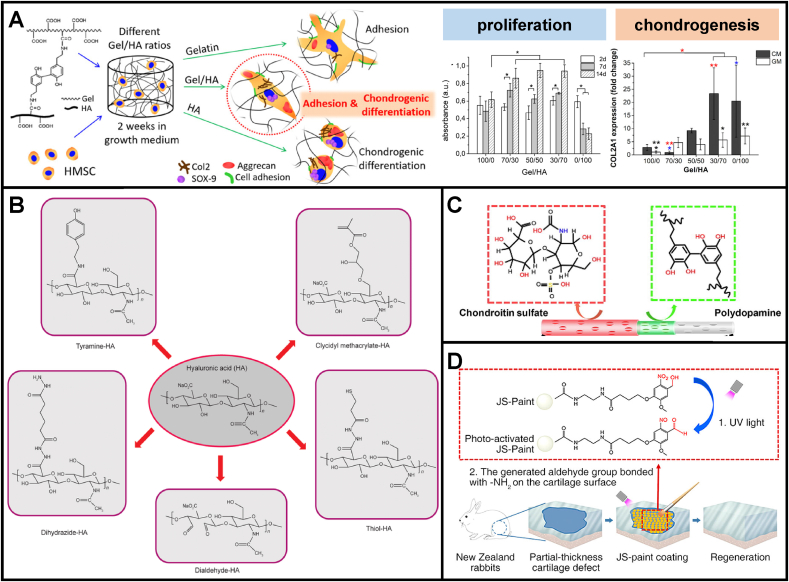
**Chemical compositions and chemical modifications of regenerative scaffolds could provide favorable biochemical cues for cell adhesion, proliferation, chondrogenesis, and ESPCs-mediated AC repair.** (A) Different chemical composition ratios of gelatin/HA hybrid hydrogels affected cell behaviors of hMSCs regarding adhesion and chondrogenic differentiation (adapted and reproduced from et al. [18], Copyright 2017, ACS). (B) A selection of chemical modifications of HA (reproduced from Ivirico et al. [43], Copyright 2017, Elsevier). (C) Chondroitin sulfate (CS) was grafted on the surface of an aligned porous fibrous membrane through PDA coatings to accelerate cartilage regeneration (reproduced from Ren et al. [19], Copyright 2019, Elsevier). (D) NB-modified SF microparticles-based tissue-adhesive paint for articular surface cartilage regeneration (reproduced from Zhang et al. [99], Copyright 2020, ACS).

A judicious selection of cartilage-mimicking biomaterials with varying tailored chemical compositions and/or (surface/interface) chemistry modifications can impact the amount or phenotype of resulting cartilage. For example, different chemical composition ratios of gelatin/HA hybrid hydrogels affected the cell behaviors of hMSCs [18] (Fig. 6A). It has been shown that pure gelatin enabled good cell adhesion without notable *in vitro* chondrogenesis of MSCs, while pure HA induced chondrogenesis without cell spreading [18]. The hydrated gelatin/HA scaffolds, particularly with more contents of HA, enhanced cell adhesion, proliferation, and chondrogenic differentiation [18]. More GAG contents were observed with elevated expression of chondrogenic markers such as sox-9, aggrecan, and collagen II [18]. Compared with naturally derived biomaterials, biologically inert synthetic biomaterials lack cell-adhesion ligands, namely cell recognition signals, and their hydrophobic nature impedes cell attachment and spreading [121]. To improve the biocompatibility of synthetic biomaterials, chemistry modifications can be utilized to generate cell–biomaterial interfaces which are beneficial for eliciting cell spreading and maintaining differentiated phenotypic expression [122]. Apart from synthetic biomaterials, natural biomaterials, for example, HA and its derivatives, have been widely utilized for EPSC-mediated AC repair [97,100,123]. The abundant –COOH and —10.13039/100002264OH functional groups support their chemistry modifications and covalent crosslinking via ester and ether linkages. The chemical functionalization of HA-based biomaterials through various functional groups has been well summarized in Ref. [43] (Fig. 6B).

Scaffold surface characteristics critically influence cell behaviors and ECM production. The hydrophilic surface has been shown to have a beneficial effect on chondrocyte activity [94]. To enhance the hydrophilic properties of the surface, hydrophilic and reactive groups such as hydroxyl, amide, and carboxyl have been introduced onto the scaffold surface by plasma treatment, ozone oxidation, aminolysis, and photo-induced grafting copolymerization of hydroxyethyl methacrylate (HEMA) or methacrylic acid (MAA) [83]. These hydrophilic groups can be used to immobilize biologically active ligands further to produce bioactive surfaces [124]. Ren et al. fabricated an aligned PLLA fiber scaffold with a biomimetic surface for accelerating cartilage repair [19] (Fig. 6C). CS was grafted on the fiber surfaces using polydopamine (PDA) as an adhesive polymeric bridge. The PLLA/PDA/CS scaffolds were implanted into cartilage defects drilled in the middle area of rabbit femoral condyles. The *in vivo* macroscopic and histological assessment results suggested that the PLLA/PDA/CS scaffolds obviously improved defects filling and hyaline AC formation compared to PLLA, PLLA/PDA scaffolds. Zhang et al. fabricated a ready-to-use tissue-adhesive joint surface paint (JS-Paint) in favor of repairing AC [99] (Fig. 6D). The JS-Paint mainly consists of N-(2-aminoethyl)-4-(4-(hydroxymethyl)-2-methoxy-5-nitrosophenoxy) butanamide (NB)-coated silk fibroin (SF) microparticles and possesses excellent properties to facilitate cell spreading, migration, and proliferation. NB-modified SF microparticles can attach directly to AC and yield a smooth layer on the surface through the photogenerated aldehyde group of NB reacting with the –NH_2_ groups of AC tissues. At six weeks post-surgery, the JS-Paint-treated groups indicated considerable improvements in repairing rabbit partial-thickness AC defects and forming smoothed surfaces. Chen et al. immobilized quercetin (QUE) on the poly (3-hydroxybutyric acid-co-3-hydroxyvaleric acid) (PHBV) scaffold through the esterification reaction to improve its bioactivity required for cartilage regeneration [125]. Chen et al. introduced carboxyl groups on the surface of PLLA nanofibers via oxygen plasma, followed by covalent grafting of cationized gelatin molecules onto the fiber surface to make it more conductive to cell attachment and spreading [83].

Additionally, surface coating of some natural macromolecules such as proteoglycans, HA, and collagen was also reported [[95], [96], [97],^126^]. Ma et al. immobilized three types of natural macromolecules (collagen, gelatin, or chitosan) on the PLLA membrane surface using a grafting-coating method to improve its biocompatibility [95]. Results confirmed that this layer of natural macromolecule attached tightly to the PLLA membrane surface. Chondrocytes cultured on this modified PLLA membranes held better cell attachment, proliferation rate, and viability. Lin et al. uncovered the improved biocompatibility and cartilage formation by HA coating on polyglycolic acid (PGA) in a rabbit model [97]. *In vitro* characterization demonstrated that HA coating enhanced cell adhesion to PGA scaffolds. This might be due to the binding between cells and the biomaterials through HA and CD44, a receptor for HA. Besides, a less inflammatory reaction was exhibited on the HA-coated scaffold *in vitro* and *in vivo* [97]. Moreover, hydrophilic coating using two or more natural macromolecules on scaffolds may have a synergistic effect. Chang et al. reported the best hydrophilicity, degradation rates, and upregulation of cell activity on HA/CS-coated PLGA scaffold compared to HA or chitosan alone [94]. Gong et al. assembled biocompatible CS and collagen I onto PLLA scaffolds layer by layer for enhancing the cell-biomaterial interactions [98]. Significant improvement in cell attachment, proliferation, cytoviability, and GAGs secretion on the PLLA/CS/collagen scaffold was achieved.

### 4.2 Exogenous chemokines, cytokines, and growth factors (CCGs)

**Fig. 7:**
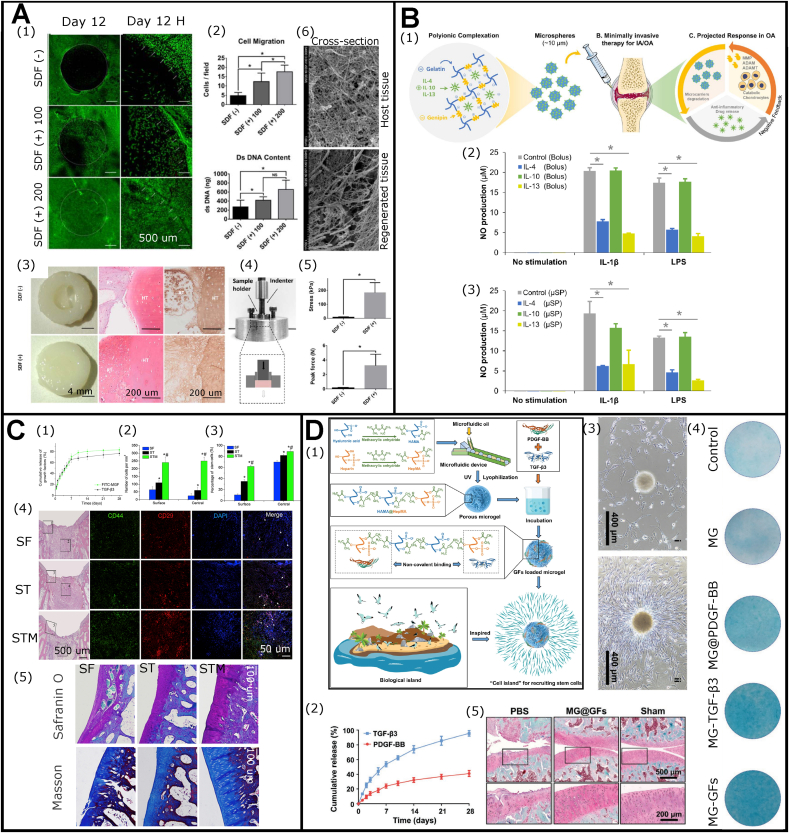
**Exogenous CCGs c**an **function as favorable biochemical cues for ESPCs-mediated AC repair.** (A) Functional repair of full-thickness bovine AC defect via homing of ESPCs by rhSDF-1α–loaded fibrin/HA composite hydrogels. Cell migration assay in response to rhSDF-1α (A1), and quantification of migrated cells and DNA contents (A2). Assessment of cartilage integration of repaired tissues in macroscopic appearance, safranin O staining, and IHC staining of Col II (A3), mechanical analysis (A4 and A5), and cross-section SEM images (A6) (Adapted and reproduced from Yu et al. [100], Copyright 2015, John Wiley and Sons). (B) Injectable microspheres for AC preservation and repair through on-demand delivery of anti-inflammatory cytokines. (B1) Graphical illustration of this study. (B2 and B3) Cytokine-loaded microspheres could modulate the inflammation status (adapted and reproduced from Park et al. [102], Copyright 2019, John Wiley and Sons). (C) MGF and TGF-β3 functionalized silk scaffolds to improve articular hyaline cartilage repair in a rabbit model. (C1) Cumulative release profiles of TGF-β3 and FITC-MGF for 28 days. (C2 and C3) Quantification of cell number of infiltration into the scaffolds and percentage of stem cells (CD29+/CD44+) at 7 days after subcutaneous implantation. (C4) Cell infiltration and multipotent stem cell identification at 7-day post-implantation in articular joint. (C5) Representative safranin O and masson's trichrome staining images of rabbit articular at 3 months after implantation (Adapted and reproduced from Luo et al. [72], Copyright 2015, Elsevier). (D) The combinational use of PDGF-BB and TGF-β3 for recruiting stem cells and repairing AC. (D1) A brief illustration of the concept of “cell island” microgels by loading with PDGF-BB and TGF-β3 as a recruiting factor and a differentiation factor of ESPCs, respectively. The injectable porous microgel was developed by photopolymerization of HAMA@HepMA blended pregel droplets generated via microfluidics. Subsequently, PDGF-BB and TGF-β3 were non-covalently incorporated into the microgels by binding heparin, creating “cell island” microgels with robust recruiting and pro-chondrogenic potentials. (D2) The release curves of TGF-β3 and PDGF-BB. (D3) The chemotaxis assay showed the cell-homing effect of the microgels. (D4) The representative alcian blue staining images indicated that microgels could promote chondrogenic differentiation *in vitro*. (D5) The representative safranin O-fast green staining images showed improved repair outcomes of MG@GFs. (Adapted and reproduced from Lei et al. [73], Copyright 2021, Wiley-VCH).

As discussed in section 2.3, inadequate endogenous CCGs would lead to failed endogenous cartilage healing. Therefore, engineering regenerative biomaterials with exogenous favorable CCGs emerged as a promising way for promoting AC repair process. Via these sufficient cues, the implant could recruit more ESPCs with cartilage regeneration capacities from adjacent niches and guide further tissue repair. Many previous studies have investigated the cell-recruiting abilities of chemokines, such as CCL2, CCL5, CCL21, CCL25, CXCL8, CXCL12, and CXCL13 [100,[127], [128], [129]]. Joutoku et al. found that exogenous CCL21 delivery to adults diminished scar-forming healing and improved hyaline-like AC formation in a rabbit OCD model. Their results showed that the CCL21/CCR7 axis might be crucial for the molecular control mechanism of juvenile AC repair, raising the possibility that agents modulating the production of CCL21 *in vivo* could enhance the quality of newly-formed cartilage among adults [130]. In a bovine explant model, Yu et al. delivered rhSDF-1α through fibrin and HA hydrogels to treat full-thickness chondral defects [100] (Fig. 7A). Using rhSDF-1α dramatically improved the recruitment of ESPCs to the defect area on day 12. It achieved significantly better cell morphology, matrix deposition, tissue ultrastructure, and mechanical properties at six weeks [100]. Besides, since acute, local inflammation and systemic inflammation appeared to hold detrimental effects on chondrogenesis and chondral healing [131], exogenous anti-inflammatory cytokines administration represented one option for AC repair. Bioresponsive gelatin microspheres loaded with IL-4, IL-10, and IL-13 as anti-inflammatory cytokines reduced inflammation and stimulated a metabolic response for AC repair [102] (Fig. 7B).

Apart from chemokines and cytokines, GFs also play crucial roles in cell proliferation and differentiation during EPSC-mediated AC repair. The study by Lee et al. demonstrated that TGFβ3-adsorbed collagen hydrogel recruited 130% more cells in the humeral regenerated AC of skeletally mature rabbits (6-month-old) compared to TGFβ3-free collagen hydrogel [132]. And thereby TGFβ3-treated group yielded hyaline cartilage regeneration and significantly greater thickness on the articular surface [132]. Similar results were reported that TGF-β1 improved the overall full-thickness cartilage defect repair in 4-month-old rabbits [133]. Nixon et al. treated critical-sized (15 mm in diameter) full-thickness cartilage defects on the lateral trochlear ridge of the distal femur of adult horses with IGF-1 fibrin clots [134]. After six months, the cartilage defects loaded with IGF-1 fibrin clots were filled with hyaline cartilage, while the IGF-1-free fibrin clots resulted in poorly organized collagen (predominantly type I) and fibroblasts. A similar effect of IGF-1 was observed in repairing partial thickness AC defects created in the knee joints of skeletally mature rabbits and mini pigs [135].

Vainieri et al. explored the *in vitro* BMSC migration under different concentrations of PDGF-BB, CCL5, and CXCL12 using a 3D spheroid-based assay and PDGF-BB was chosen as the most promising chemotactic factor [136]. *In vivo* data indicated that both hydrogels strengthened ESPC infiltration and supported a favorable microenvironment for producing neocartilage using an osteochondral explant model implanted subcutaneously in athymic mice. Of note, these processes were best supported in fibrin-HA hydrogels without PDGF-BB [136]. Additionally, combinational utilization of CCGs exhibited some advantages in eliciting its maximal chemotactic performance. Luo et al. combined mechano growth factor (MGF) and TGF-β3 into silk scaffolds for AC repair in a rabbit model [72] (Fig. 7C). This combination significantly increased cell recruitment ability *in vitro*. The MGF/TGF-β3-treated group produced more cartilage-like ECM and less fibrillar collagen than MGF- or TGF-β3-treated group [72]. Lei et al. fabricated a PDGF-BB and TGF-β3 loaded HAMA and heparin blend microgel for AC repair in a rat model [73] (Fig. 7D). The studies showed that the microgel could improve the migration ability of ESPCs and recruit them from surrounding niches by releasing PDGF-BB. Via using HA, the “cell island” microgels provided an amenable microenvironment for cell attachment and spreading. Furthermore, the “cell island” microgels induced chondrogenic differentiation of the recruited ESPCs through releasing TGF-β3 and presented an excellent repairing potential for cartilage. To date, all these strategies are on preclinical stages and much more efforts are needed for their translation.

### 4.3 Mineral ions

**Fig. 8:**
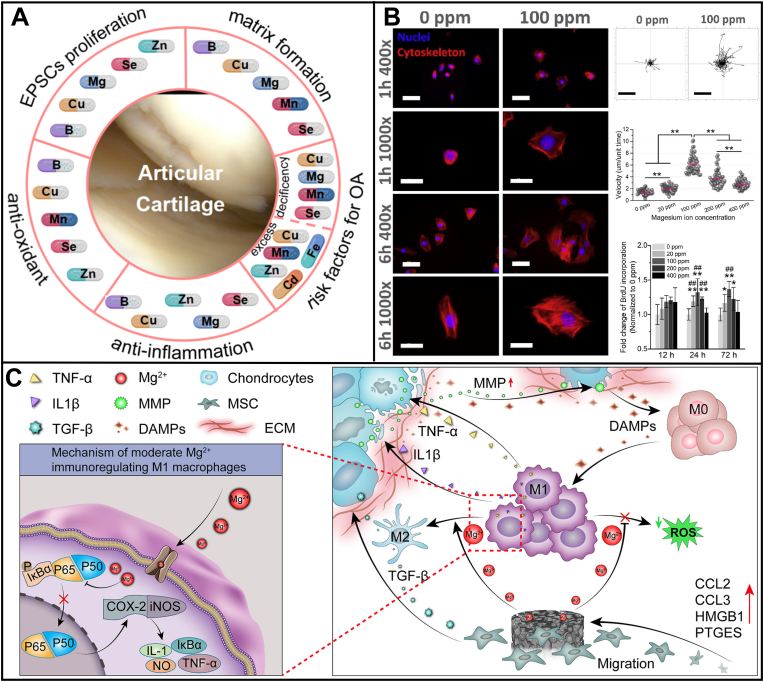
**The crucial roles of mineral ions in the example of Mg**^**2+**^**are to regulate cell behaviors of stem/progenitor cells (SPCs) and enhance cartilage repair.** (A) Mineral ions play irreplaceable roles in regulating cellular behavior and promoting cartilage healing. (B) Effects of different Mg^2+^ concentrations on cell adhesion, migration, and proliferation *in vitro* (reproduced from Shen et al. [142], Copyright 2021, Elsevier). (C) Via the controlled release of Mg^2+^, Mg-based scaffolds could enhance chondrogenesis and eliminate the destructive effects of activated macrophages on chondrocytes (reproduced from Zhao et al. [21], Copyright 2022, Elsevier).

As cofactors of enzymes or immunomodulators, many mineral ions (e.g. Zinc (Zn), boron (B), selenium (Se), cobalt (Co), calcium (Ca), copper (Cu), magnesium (Mg), manganese (Mn) ions) are involved in the proliferation, attachment, and differentiation of ESPCs, matrix formation, anti-inflammation and tissue homeostasis [137] (Fig. 8A). Optimized mineral ions can impart these biochemical cues to implants for enhancing ESPCs-mediated AC repair. Co ions are simulated hypoxia inducers, and the hypoxia-induced transcriptional profile plays a vital role in chondrogenic differentiation [138]. The incorporation of Co ions into alginate scaffolds could support chondrogenesis by mimicking the hypoxia environment following a dose-dependent manner [139,140]. Lv et al. incorporated Co or Ca ions into an injectable GelMA-sodium alginate (SA) hydrogel to promote cartilage formation in an eight-week-old male rat model [140]. After eight weeks, the empty defects were filled with fibrous tissues, while the GelMA-SA-Ca group obtained a better fill-in with a mixture of cartilage-like and fibrous tissues. In comparison, the GelMA/SA-Co group achieved the best cartilage repair with a similar structure to native cartilage. Cu ions could enhance the chondrogenesis of MSCs by promoting the MSCs' cytoskeleton change and up-regulating the chondrogenic gene expression [106,141]. Adding Cu into a porous alginate scaffold improved cartilage formation in adult male mice models [141]. Shimaya et al. reported that Mg ions enhanced cell adherence and cartilage formation by endogenous rabbit S-MSCs through integrins *in vivo* [105]*.* Further study revealed the effects of different Mg ion concentrations on cell adhesion, migration, and proliferation *in vitro* [142] (Fig. 8B). Fluorescent staining showed that medium containing 100 -ppm Mg ions boosted cell-substratum adhesion, and cells in this group showed larger and polygonal cell morphologies. In contrast, the 0 ppm Mg^2+^-treated group exhibited delayed cell-substratum adhesion. The 100 ppm Mg^2+^-treated group demonstrated the highest cell migration velocity and cell proliferation. The study from Zhao et al. develop a porous Mg-Nd-Zn-Zr alloy scaffold coated with PDA and validated their cytocompatibility and impacts on immunomodulation for AC repair [21] (Fig. 8C). This study revealed the advantageous potential of Mg-based implants to expedite chondrogenesis by controlled release of Mg^2+^ in addressing the destructive effect of activated macrophage polarization on chondrocytes. The commercial product MaioRegen also contains Mg in the lower layer of Mg-HA as favorable external biochemical cues to recruit ESPCs and guide AC repair.

### 4.4 Chondroinductive/chondroconductive peptides

**Fig. 9:**
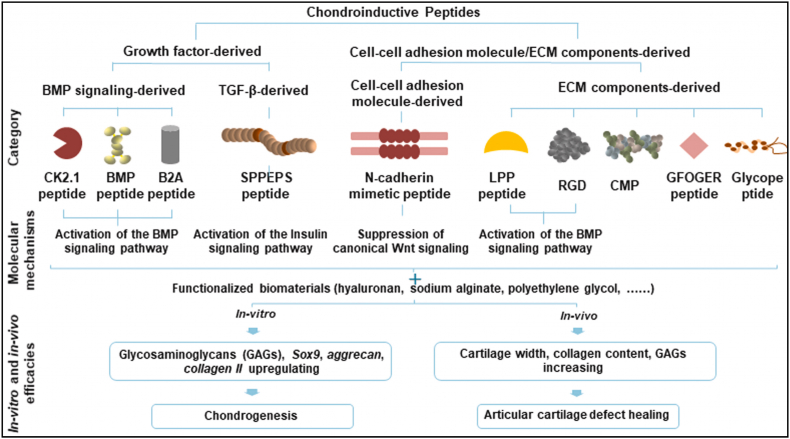
**Chondroinductive/chondroconductive peptides c**an **boost the chondrogenesis of ESPCs and AC repair.** Growh factor- and cell-cell adhesion molecule/ECM components-derived peptides can activate distince molecular mechanisms. CMP: collagen mimetic peptide; LPP: link protein N-terminal peptide. (Reproduced from Zhu et al. [143], Copyright 2021, Elsevier).

Peptides are a particular category of bioactive substances which can be engineered into/onto biomaterials to serve as chondroinductive/chondroconductive biochemical cues [143]. Compared with proteinous GFs, chemically synthesized peptides are more advantageous in quantity, efficiency, and purity and can be easily modified for improved functionalities. Other than direct mixture and self-assembly, the chemical conjugation approaches involve Michael addition and temperature- or UV-induced crosslinking. Therefore, these peptide-functionalized biomaterials showcase great promise in ESPCs-mediated AC repair. Typically, chondroinductive/chondroconductive peptides can be categorized into two types: GF-derived peptides (e.g. CK2.1, BMP, B2A, and SPPEPS peptide) and cell-cell adhesion molecules/ECM components-derived peptides (e.g. N-cadherin memetic peptide, LPP peptide, RGD, CMP, GFOGER, and Glycope peptide) [143] (Fig. 9). They mainly function through BMP, ERK, Smad, TGF-β, and Wnt signaling pathways (indicated in Fig. 2) to upregulate the Sox 9, Aggrecan, and Collagen Ⅱ expression and GAG contents for enhanced AC defect healing. Most peptides are chondroconductive instead of chondroinductive. For some peptides (i.e. 100 nM CK2.1) induce chondrogenesis more efficiently both *ex vivo* (micromass model) and *in vivo* (mice knee AC defects) compared with 40 nM BMP-2 proteins [144]. Moreover, this peptide results in no or much less hypertrophy and mineralization [144], which is of paramount importance for maintaining the hemostasis of neo-cartilage. Liu et al. fabricated CK2.1-coated β-glycerophosphate chitosan (CK2.1@GC) composite scaffolds for AC repair in a rabbit model through the recruitment and induced chondrogenesis of ESPCs [23]. SPPEPS, a TGF-β3-derived peptide, seemed to be more chondroconductive rather than chondroinductive due to its very mild potency in inducing chondrogenesis and it could only enhance *in vitro* collagen II expression [145]. Future research should concentrate on the (bio)design, (bio)fabrication, and assessment of more potent chondroinductive peptides and peptides-functionalized scaffolds with *in vivo* efficacies to facilitate their clinical translation.

### 4.5 Small molecule compound drugs

**Fig. 10:**
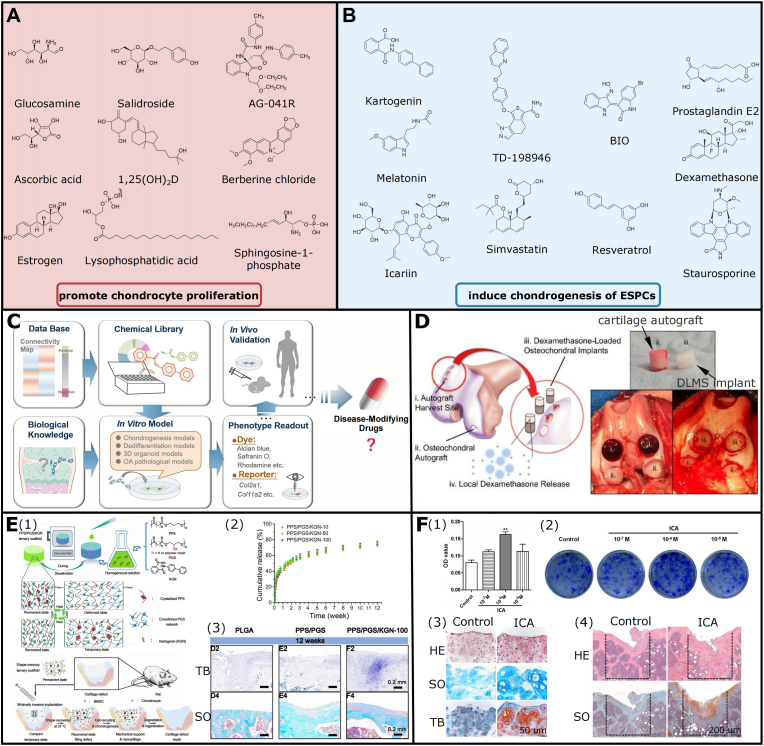
**Small molecule compounds c**an **function as favorable biochemical cues for ESPCs-mediated AC repair.** (A, B) Small molecule compounds could support chondrocyte proliferation and chondrogenesis of progenitor/stem cells (adapted and reproduced from Li et al. [146], Copyright 2020, Elsevier). (C) State-of-the-art screening strategies of small molecular drugs for AC repair (reproduced from Chen et al. [148], Copyright 2021, Springer Nature). (D) Improved AC and subchondral bone repair by a PLGA microsphere-embedded agarose scaffold via sustained delivery of dexamethasone (reproduced from Stefani et al. [113], Copyright 2020, Elsevier) (E) A cell-free strategy for AC repair by biofunctionalized chondrogenic shape-memory ternary PPS/PGS/KGN scaffolds. (E1) Schematic diagram of this study. (E2) KGN release curves of PPS/PGS scaffolds with different original KGN contents. (E3) The representative toluidine blue and safranin O staining images of AC repair in different groups at 12 weeks (adapted and reproduced from Xuan et al. [149], Copyright 2020, Elsevier). (F) Icariin could activate HIF-1α in chondrocytes and promote AC repair. (F1) MTT assay for cell viability of chondrocytes and (F2) colony formation assay for chondroprogenitor cells indicated that Icariin could promote chondrocyte proliferation. (F3) The representative HE, safranin O, and toluidine blue staining images showed that icariin promotes chondrogenesis in the alginate-chondrocyte 3D culture system. (F4) The representative HE and safranin O staining images showed icariin could enhance AC regeneration in a mouse OCD model (adapted and reproduced from Wang et al. [150], Copyright 2016, PLOS).

Small molecule compounds allow for a simple and efficacious approach to enhance chondrocyte proliferation, cell phenotype maintenance, and chondrogenesis of SPCs [[146], [147], [148]]. Accordingly, regenerative biomaterials functionalized with appropriate small-molecule drugs represent a feasible option to enhance ESPCs-mediated AC repair. They can be summarized in two options: (1) promoting chondrocyte proliferation (e.g. Glucosamine, Ascorbic acid, Estrogen, Salidroside, 1,25(OH)_2_D_3_, Lysophosphatidic acid, AG-041R, Berberine chloride, and Sphingosine-1-phosphate); and (2) inducing chondrogenesis (e.g. KGN, Melatonin, Icariin, TD-198946, Simvastatin, BIO, Resveratrol, Prostaglandin E2, Dexamethasone, and Staurosporine) [146] (Fig. 10A and B). They mainly function via TGF-β, MAPK, Wnt, IGF, and IHH signaling pathways indicated in Fig. 2. And they hold several superiorities in rapid, reversible, and dose-dependent bio-effect, chemical modification, large-scale production, cost-effectiveness, and straightforward administration [[146], [147], [148]]. Two disadvantages of small molecule functionalization are multiple targets and unexpected toxicity, impeding their further applicability [[146], [147], [148]]. Of note, currently only glucosamine, icariin, and estrogen have been used in cartilage treatment clinically [146]. The emerging technologies of streamlined high-throughput drug screening platforms bring new hopes, and they can simplify and accelerate the research and development of targeted small molecule compounds, expediting their ultimate translation processes (Fig. 10C).

Currently, most of the above-mentioned small molecule compound drugs are still in preclinical stages and have achieved desirable animal results to some degree. For instance, Stefani et al. established a sustained delivery system with low-dose dexamethasone by a PLGA microsphere-embedded agarose implant to markedly enhance AC repair in dogs [113] (Fig. 10D). The controlled presentation of dexamethasone (up to 99 days) exhibited dual pro-anabolic and anti-catabolic effects, both facilitating tissue integration whereas also mitigating excess inflammation [113]. KGN and icariin could also function as chondrogenic factors. Xuan et al. (bio)fabricated a chondrogenic and physiological-temperature-triggered shape-memory ternary scaffold for cell-free AC repair in a rat model [149] (Fig. 10E). Within the scaffold, poly (glycerol sebacate) (PGS) networks supported shape recovery and elasticity properties; crystallized poly (1,3-propylene sebacate) (PPS) served as switchable phase; and incorporated KGN ensured the scaffold with pro-chondrogenic ability. The *in vitro* scaffold degradation and cumulative release curve indicated that the sustained release of KGN could last at least 12 weeks [149]. The *in vivo* studies suggested that the PPS/PGS/KGN scaffolds enhanced neocartilage regeneration in the absence of exogenous GFs and seeded cells [149]. Besides, icariin could activate HIF-α in chondrocytes and promote AC repair [150] (Fig. 10F). The data showed that Icariin may suppress prolyl hydroxylase domain (PHD) activity via competing for cellular iron ions and it might act as an HIF-1 activator to enhance AC regeneration by controlling chondrocyte differentiation, proliferation, and tissue integration [150].

### 4.6 Gene targeting factors and EVs

**Fig. 11:**
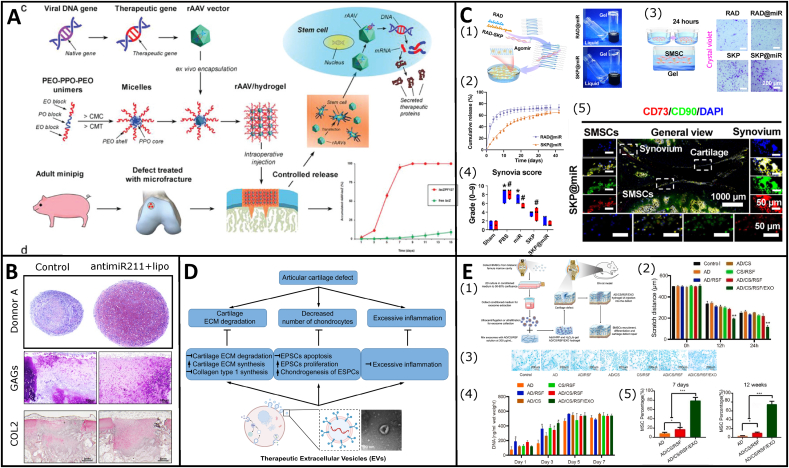
**Gene targeting factors and EVs could function as favorable biochemical cues to enhance ESPCs-mediated cartilage repair.** (A) PEO–PPO–PEO poloxamers-based thermosensitive hydrogel loaded with rAAV for efficient gene therapy of AC defects (reproduced from Madry et al. [118], Copyright 2020, Wiley). (B) Hydrogel-based delivery of antimiR-221 improved chondral defect repair through ESPCs (reproduced from Lolli et al. [24], Copyright 2019, Elsevier). (C) Hydrogel-based delivery of miR-29b-5p to recruit ESPCs for cartilage repair by suppressing senescence in a rat model. A brief illustration of the (C1) fabrication of hydrogel-miRNA constructs. (C2) Cumulative release profiles of agomir-29b-5p at 37 °C. (C3) Transwell assay to monitor ESPCs recruitment *in vitro*. (C4) Synovia scores, and (C5) immunofluorescence staining to show SKP@miR induced ESPCs recruitment and enhanced chondrogenic differentiation *in vivo* (adapted and reproduced from Zhu et al. [115], Copyright 2022, AAAS). (D) Therapeutic EVs as promising cell-free strategies for AC regeneration (adapted and reproduced from Amsar et al. [159], Copyright 2022, Future Science). (E) Injectable mussel-inspired highly adhesive hydrogel with exosomes for recruiting ESPCs and repairing AC defect. (E1) Schematic illustration of this study. (E2) Scratch distance assay to show the migration and infiltration of BMSCs *in vitro*, (E3) Alcian staining to show the effect of pro-chondrogenic differentiation on BMSCs *in vitro*, (E4) DNA concentration analysis to show the improved proliferation effect on BMSCs *in vitro*, and (E5) Semi-quantitative analysis of stem cell percentage to show the enhanced migration and infiltration effect on ESPCs *in vivo* (adapted and reproduced from Zhang et al. [50], Copyright 2021, Elsevier and Fan et al. [163], Copyright 2022, MDPI).

Numerous gene targeting factors (e.g. siRNA, mRNA, miRNA, and CRISPR/Cas9) can be engineered into regenerative implants for ESPCs-mediated cartilage repair. Advanced biomaterial-guided delivery of gene vectors is an emerging and highly desirable therapeutic option for AC repair, enabling the spatiotemporally controlled and minimally invasive delivery of vectors and minimizing intra-articular vector spread and potential loss of the therapeutic gene products. Madry et al. fabricated an injectable and thermosensitive PEO–PPO–PEO hydrogel system, capable of repairing full-thickness chondral defects in a minipig model through the controlled release of a therapeutic rAAV vector overexpressing the chondrogenic sox 9 transcription factor [118] (Fig. 11A). Additionally, miRNAs can also modulate gene expression via inhibiting translation or triggering mRNA degradation, affecting cell behaviors and even cell fate [151]. miRNAs expression profiles differ during AC development and MSC chondrogenesis, indicating their significant role during cartilage healing. Lolli et al. reported miR-221 as a novel anti-chondrogenic miRNA, and silencing miR-221 in human BM-MSCs could improve chondrogenesis [152,153]. Lolli et al. further silenced miR-221 in ESPCs by fibrin/HA hydrogels loaded with locked nucleic acid (LNA)-microRNA inhibitors via non-viral transfection [24]. AntimiR-221 significantly promoted chondrogenesis and AC repair in a semi-orthotopic model of bovine osteochondral tissues implanted subcutaneously in nude mice [24] (Fig. 11B). Even under an inflammation environment, hydrogel-based delivery of miR-29b-5p could stimulate to recruit ESPCs for cartilage repair by suppressing senescence in an OA rat model [115] (Fig. 11C). Besides, some *in vitro* experiments confirmed the pro-migratory effects of miRNAs, such as miR-10b [154] and antimiR-375 [155]. Apart from miRNAs, aptamers (single-stranded DNA or RNA) with unique tertiary structures could bind specifically with cognate molecular targets [156]. Wang et al. showed that an Apt19S-functionalized bilayer scaffold could recruit BM-MSCs and support cell adhesion both *in vitro* and *in vivo* for AC repair macroscopically and histologically in a rabbit model [117].

Furthermore, actively released by a variety of cells, EVs are small membrane-enclosed particles [157]. The mRNAs, miRNAs, and DNA carried in EVs can potentially be transferred to neighboring cells, inducing persistent and prolonged genetic reprogramming and modifying their phenotype as well as the microenvironment [158]. On the basis of biogenesis, size, and content, EVs can be divided into three categories: exosomes (40–200 nm), microvesicles/shedding particles (50–1000 nm), and apoptotic bodies (500–2000 nm) [157,159]. Recently, EVs have been proposed as emerging tools for restoring joint homeostasis and improving AC regeneration [159] (Fig. 11D). As a result of heterogeneous contents, selecting suitable parental cells and proper therapeutic methods are indispensable for targeting treatment of AC defects [159]. Loading EVs by biomaterials optimizes their effectiveness for cartilage regeneration. A promising tissue patch for rabbit AC regeneration was constructed by Liu et al. through the integration of HA-NB/Gelatin hydrogel glues and stem cell-derived exosomes [160]. Zhang et al. fabricated an injectable mussel-inspired highly adhesive hydrogel for the local delivery of exosomes [50] (Fig. 11E). Exosomes released from the AD/CS/RSF/EXO hydrogel maintained their structures and bioactivities. These released exosomes largely contributed to the recruitment and inflation, proliferation, and differentiation of BM-MSCs *in vitro*, and improved rat AC repair with mature ECM remodeling by recruiting ESPCs *in vivo* [50]. Shen et al. developed a silk hydrogel loaded with hypoxia preconditioned MSCs-derived EVs to repair AC through the miR-205–5p/PTEN/AKT pathway [161]. The hypoxia preconditioned EVs significantly boosted the proliferation, migration, and anabolism of chondrocyte cells and anti-inflammatory effects [161]. This was in accordance with a study by Xue et al. [162]. Despite the promising preclinical results, more in-depth research should focus on addressing the related problems, such as high homogeneous EVs and large-scale production, to facilitate their clinical application.

### 4.7 Anti-inflammatory and immunomodulatory agents

**Fig. 12:**
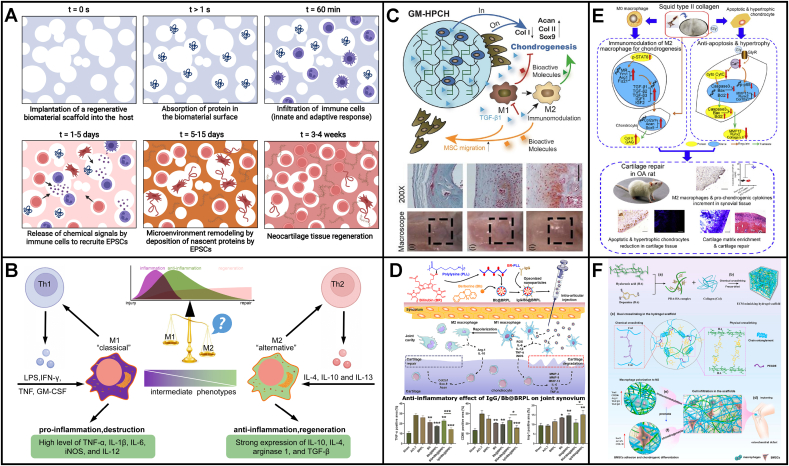
**Engineering regenerative biomaterials with optimized biochemical cues with good anti-inflammatory and immunomodulatory effects for ESPCs-mediated AC repair.** (A) Immune response following the implantation of regenerative biomaterials (created by BioRender). (B) The delicate polarization shift balance between pro-inflammatory M1 and regenerative M2 macrophages and their distinct effects on cartilage healing. (C) The thermosensitive photocrosslinkable TGF-β1-loaded composite hydrogel facilitated AC repair through immunomodulating macrophages, recruiting ESPCs, and expediting chondrogenesis (reproduced from Ji et al. [25], Copyright 2020, Ivyspring). GM-HPCH: glycidyl methacrylate-modified hydroxypropyl chitin. (D) A trapping strategy for AC regeneration via opsonized nanoparticles to selectively target M1 macrophage and promote M1-to-M2 polarization shift (reproduced from Kou et al. [167], Copyright 2022, Elsevier). (E) Under degenerative OA conditions, AC repair was significantly enhanced by squid type II collagen via inhibiting apoptosis and hypertrophy of chondrocytes and immunomodulating activation of M2 macrophages (reproduced from Dai et al. [120], Copyright 2018, Elsevier). (F) A cell- and GF-free AC repair strategy based on the mussel-inspired ECM-mimicking hydrogels with excellent cell affinity and immunomodulation capability (reproduced from Gan et al. [169], Copyright 2022, Elsevier).

The inflammation phase of AC repair is pivotal since it orchestrates all the following biological activities. A cascade of reactions is triggered immediately after biomaterials implantation, including a layer of proteins from the surrounding vasculature adsorbs onto the biomaterial surface, infiltration adherence of various immune cells (e.g. platelets, neutrophils, monocytes, and macrophages), the release of physicochemical signals by immune cells to recruit ESPCs, microenvironment remodeling by deposition of nascent proteins by ESPCs, and neocartilage formation (Fig. 12A). At present, a critical mode toward the better regeneration of AC is supported by implant-mediated immunomodulation [164]. Studies have indicated that biomaterials can significantly impact the polarization of macrophages and T cells, which hold extensive cell crosstalk with ESPCs [165]. So far, the polarization shift from pro-inflammatory (M1) to anti-inflammatory macrophage (M2) phenotypes has been increasingly investigated to alleviate excessive inflammation, which can be applied to the biomaterial design principles based on the macrophage-mediated immunomodulatory healing of AC [165] (Fig. 12B). Thus, many immunomodulation cues can be engineered into regenerative scaffolds to achieve this goal based on the macrophage polarization shift. Yuan et al. fabricated the hydroxypropyl chitin (HPCH) hydrogel and confirmed its function in activating inflammatory responses and recruiting endogenous macrophages to support a suitable inflammation microenvironment [166]. Following this pattern, Ji et al. synthesized a thermosensitive photocrosslinkable glycidyl methacrylate-modified HPCH hydrogel (GM-HPCH) loaded with TGF-β1 [25] (Fig. 12C). The *in vivo* and *in vitro* studies revealed that a GM-HPCH + TGF-β1 treatment markedly shifted the recruited macrophages from M1 to M2 [25]. The composite hydrogel boosted the expression of chondrogenic genes and the migration of BM-MSCs and achieved superior cartilage healing [25]. To treat OA, Kou et al. prepared artificial opsonized nanoparticles (IgG/Bb@BRPL) which can selectively target M1 macrophages to repolarize M1-to-M2 by reactive oxygen species scavenging and NF-κB pathway deactivation [167] (Fig. 12D). Dai et al. developed a squid-derived collagen II (SCII) scaffold that could suppress the pro-inflammatory macrophages via inhibiting the STAT1 signal for cartilage lesions [120,168] (Fig. 12E). SCII scaffolds induced the M1 macrophage polarization into the M2 phenotype and promoted macrophages to express pro-chondrogenic genes as well as the production of Collagen II and GAGs *in vitro* [120]. Inspired by mussel, Gan et al. fabricated a ECM-mimicking composite hydrogel with high cell infiltration and immunomodulation capability for GFs-free AC repair in a rabbit model [169] (Fig. 12F).

Apart from macrophages, regenerative scaffolds could also impact other immune cells’ phenotypes to modulate AC repair. For example, the recruitment of neutrophils is necessary for the onset of inflammation, but sustained neutrophils might result in long-term chronic pro-inflammation and then failed repair. Engineered anti-inflammatory and immunomodulatory cues of biomaterials have significant implications for neutrophils' activation and function [37]. Hoemann et al. proved that chitosan–glycerol phosphate/blood implants could attract more neutrophils *in vitro* and *in vivo* compared to whole blood clots, though both released a similar profile of chemotactic factors (CCL2, CXCL8, PDGF-BB) [170]. And after eight weeks *in vivo*, more uniform and integrated cartilage tissue was observed in the trochlear cartilage defects treated with chitosan–glycerol phosphate/blood implants, indicating the potential roles of neutrophils in AC repair [170]. Such studies highlighted the significance of investigating biomaterials in the framework of immunomodulation of immune cells (i.e. macrophages) and their repair responses in ESPCs-mediated AC regeneration.

### 4.8 Spatiotemporal delivery/release modalities

**Fig. 13:**
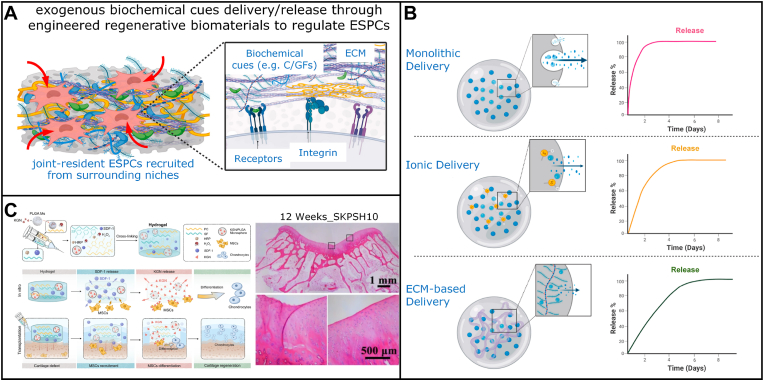
**Engineering regenerative biomaterials as spatiotemporal delivery/release modalities of various biochemical cues to guide ESPCs for cartilage repair.** (A) Novel regenerative scaffold-based DDS loaded with favorable exogenous biochemical cues to recruit ESPCs from surrounding niches for ESPCs-mediated AC repair (adapted and reproduced from Gresham et al. [69], Copyright 2021, Elsevier). (B) Representative release profiles of GFs-based DDS. Each profile (monolithic, ionic, and ECM-based delivery) is graphically illustrated to reveal its releasing mechanism (reproduced from Gresham et al. [69], Copyright 2021, Elsevier). (C) Sequential delivery of SDF-1/KGN by injectable PC/SF composite hydrogels for spatiotemporal regulation of endogenous cells to improve AC repair (reproduced from Dong et al. [177], Copyright 2021, Springer Nature).

Engineered regenerative constructs might address the challenges of traditional drug delivery approaches by improving biochemical cues' delivery, retention, targeting, and bioactivity [171]. To closely recapitulate the innate repairing cascades, controlled sequential release of exogenous bioactive factors to the defect site has been established to enhance ESPCs-mediated cartilage repair [28,113,171]. The (bio)design and (bio)fabrication of innovative scaffold-based drug delivery systems (DDS) offered new possibilities for sophisticated release kinetics of various bioactive substances, including specific drugs, CCGs, mineral ions, small molecule compounds, peptides, anti-inflammatory agents, gene targeting factors and EVs (Fig. 13A).

By intra-articular injection-based local delivery, free drugs cannot exist for a very long duration (mostly within hours or days) due to joint clearance. As soon as being injected into joints, drugs get into synovial fluid with a rapid physiological turnover. When it comes to a short therapeutic time frame, some doctors try to reduce the injection frequency, aiming to prolong the drug's residence. Encouragingly, novel DDS technologies demonstrate tremendous potential for addressing the above-mentioned problems. A modest improvement in intra-articular presence can significantly influence the drug's exposure time. Ionic and ECM-based GFs delivery leverage the interaction of positively charged proteins with negatively charged substrates for longer effective days, compared with monolithic carriers releasing GFs by diffusion [69] (Fig. 13B). Current efforts are mainly focused on strengthening these bioactive factors' localization, retention, bioactivity, delivery, and targeting [171]. Through physical adsorption, direct blending, surface grafting, drop casting, chemical immobilization, covalent bonding, coaxial electrospinning, and microparticles incorporation, bioactive factors could be combined with biomaterials [172]. For instance, SDF-1α/TGF-β1 can be physically absorbed and sustained release from a SF-porous gelatin scaffold [90]. The release kinetics of SDF-1α/TGF-β1 encapsulated within hydrogels were controllable via hydrogel properties such as mesh size [173]. However, physical absorption or encapsulation within regenerative scaffolds has shown some disadvantages, such as unexpected burst release at an early time point. Covalently binding to the scaffold surfaces provides accurate and controllable spatiotemporal delivery and avoids burst release. Lee et al. achieved the directional PDGF-AA release using catecholamine adhesion chemistry to develop robust interfacial adhesion covalently, which greatly promoted the recruitment of ESPCs and cartilage repair [174]. However, poorly controlled conjugation can negatively influence the conformation and biological activity of GFs. For example, when BMP-2 was covalently bound to the surface of a PCL scaffold and the conjugation provided a sustained release of BMP-2, negligible alkaline phosphatase deposition and less tissue ingrowth were observed compared to the one by physical absorption, which had a small burst release of BMP-2 [175]. Besides, binding and mimetic peptides of biomaterials could be designed to interact with specific regions of CCGs. This process could be utilized to manipulate the releasing profile of DDS.

However, the release of biochemical cues by regular DDSs only sustained for a relatively short period, which is insufficient for long-term and complete AC regeneration [28]. Innovative DDSs-based strategies, such as nano-carriers, liposomes, and micelles were extensively studied to achieve a prolonged release or even penetration into cartilage [26]. Zhang et al. reported an advanced all-silk-derived sequential DDS through incorporating the tunable drug-loaded SF nanospheres into a SF porous matrix, which could provide a sustained and relatively slow release paradigm of KGN longer than one month [176]. Dong et al. fabricated a chitosan/SF hydrogel with PLGA microspheres to deliver KGN and SDF-1 simultaneously [177] (Fig. 13C). PLGA microspheres were evenly spread within the chitosan/SF hydrogel, permitting the sequential release of those two drugs. SDF-1 and KGN served as recruiting factors and chondroinductive factors, respectively. This special (bio)design markedly improved the cell homing and chondrogenic differentiation of ESPCs *in vitro* and *in vivo* and cartilage repair in a rabbit model. Theoretically, the delivery/release profiles of different bioactive substances can meet the timeline of diﬀerent AC healing stages via taking advantage of the inherent properties of distinct biomaterials and spatially organized components. Unfortunately, an ideal DDS that can sequentially release bioactive factors in every repairing stage for enhancing AC restoration has rarely been reported. Much more efforts are required to discover an optimal DDS and advance its clinical translation.

## 5 Concluding remarks and perspectives

Compared with endogenous cartilage healing (i.e. bone marrow stimulation techniques) and *in vitro* manipulated cell-based treatments, cell-free regenerative scaffolds-based strategies exhibited many advantages, such as less donor-site morbidities, the absence of cell selection, delivery, viability, and phenotypic stability issues, the timing of pre-treatments, regulatory issues, and low costs. Meanwhile, several acellular commercial products, such as Agili-C™ and MaioRegen Chondro+ have been approved by regulatory bodies, reinforcing our translational determination and direction. Therefore it is time to highlight and summarize the proposed strategies based on the synergistic effects of endogenous cells and biochemical cues from regenerative scaffolds for cartilage repair. Of note are the target groups based on EPCSs-mediated AC repair strategies are young patients with cartilage defects due to trauma and may not be suited for patients with progressed OA and older patients. The question now is how to stimulate joint-resident ESPCs effectively and adequately for *in vivo* cartilage defect repair. So far, many methodologies have been adopted to (bio)design and (bio)fabricate novel regenerative constructs with optimized biochemical cues and spatiotemporal delivery/release modalities, achieving desirable outcomes to some degree. After implantation, these biomaterials can interact with the adjacent tissues through these biochemical signals, which alter local tissue microenvironments by modulating the immune system and controlling the kinetics and degree of healing by activating and recruiting a large population of join-resident ESPCs infiltration, guiding their mobilization, proliferation, chondrogenesis, matrix deposition, and remodeling to generate hyaline-like cartilage eventually. This review may provide a basic summary of these biochemical cues for the successful activation and maintenance of ESPCs-mediated AC repair.

Based on the literature review, at present, we cannot conclude which biochemical cue or combination has the most robust effect due to the lack of standardized comparison in the preclinical studies. The preclinical assessments use different animal models, defect locations, defect sizes, histological/CT methods, and scoring/semi-quantification systems. No consensus of standardized and streamlined preclinical evaluation protocols and procedures exist currently to compare different engineered biochemical cues. It is envisioned that more standardized preclinical studies are performed allowing to better compare the results between labs and clinics. The cartilage tissue engineering community has the obligation to tackle this issue soon to facilitate the ultimate translation of acellular regenerative biomaterials with optimized and robustly engineered biochemical cues for cartilage repair via initiating and magnifying the roles of ESPCs. Besides, future research work shall address the following aspects: (1) to investigate the roles of endogenous cells (e.g. different immune cells and joint-resident progenitor/stem cells): and the underlying molecular mechanisms of ESPCs-mediated AC repair; (2) to uncover how these exogenous biochemical cues influence exogenous cell behaviors *in vivo* and how to manipulate and control these biochemical cues properly *in vivo* to satisfy multiple demands of different healing stages; (3) to solve the technical barriers of combining biochemical cues with cartilage-mimicking regenerative scaffolds thoroughly; (4) to innovate more advanced methodologies (i.e. 3D-(bio)printing technology) to (bio)design and (bio)fabricate regenerative implants with excellent biophysical and biochemical properties; (5) to improve the delivery, retention, targeting, and bioactivity of exogenous biochemical cues within the joint via spatiotemporal scaffold-based DDS. Although the journey from bench to bedside is very draining, the multidisciplinary approaches involving material scientists, biologists, engineers, and clinicians seem to be a winning strategy to speed up the translational procedure. Our proposed solutions may represent silver linings for cartilage regeneration.

## CRediT authorship contribution statement

**Liangbin Zhou:** Conceptualization, Writing – original draft – revision & editing, Figure – drawing & revision & editing. **Jietao Xu**: Writing – original draft – revision & editing. **Andrea Schwab:** Writing – revision & editing. **Wenxue Tong:** Writing – revision & editing. **Jiankun Xu:** Writing – revision & editing. **Lizhen Zheng:** Writing – revision & editing. **Ye Li:** Writing – revision & editing. **Zhuo Li:** Writing – revision & editing. **Shunxiang Xu:** Writing – revision & editing. **Ziyi Chen:** Writing – revision & editing. **Zou Li:** Writing – revision & editing. **Xin Zhao:** Writing – revision & editing. **Gerjo VJM van Osch:** Supervision, Writing – revision & editing. **Chunyi Wen:** Supervision, Writing – revision & editing. **Lin Qin:** Conceptualization, Supervision, Writing – revision & editing, Funding acquisition.

## Declaration of competing interest

The authors declare that they have no known competing financial interests or personal relationships that could have appeared to influence the work reported in this paper.
